# First Report on the Finding of *Listeria mnocytogenes* ST121 Strain in a Dolphin Brain

**DOI:** 10.3390/pathogens9100802

**Published:** 2020-09-28

**Authors:** Yann Sévellec, Marina Torresi, Benjamin Félix, Féderica Palma, Gabriella Centorotola, Stefano Bilei, Matteo Senese, Giuliana Terracciano, Jean-Charles Leblanc, Francesco Pomilio, Sophie Roussel

**Affiliations:** 1 Maisons-Alfort Laboratory for Food Safety, *Salmonella* and *Listeria* Unit, University of Paris-Est, French Agency for Food, Environmental and Occupational Health & Safety (ANSES), 94700 Maisons-Alfort, France; yann.sevellec@anses.fr (Y.S.); benjamin.felix@anses.fr (B.F.); federicapalma8@gmail.com (F.P.); jean-charles.leblanc@anses.fr (J.-C.L.); 2 Italian National Reference Laboratory for *Listeria monocytogenes*, Istituto Zooprofilattico Sperimentale dell’Abruzzo e Molise “G. Caporale”, IZSAM, 64100 Teramo, Italy; m.torresi@izs.it (M.T.); g.centorotola@izs.it (G.C.); f.pomilio@izs.it (F.P.); 3 Istituto Zooprofilattico Sperimentale del Lazio e della Toscana M. Aleandri, 00178 Rome, Italy; stefano.bilei@izslt.it (S.B.); matteo.senese@izslt.it (M.S.); giuliana.terracciano@izslt.it (G.T.)

**Keywords:** *Listeria monocytogenes*, ST121, dolphin, wildlife, food, genomic comparison

## Abstract

*Listeria monocytogenes* (*Lm*) is a ubiquitous bacterium that causes the foodborne illness, listeriosis. Clonal complexes (CC), such as CC121, are overrepresented in the food production industry, and are rarely reported in animals and the environment. Working within a European-wide project, we investigated the routes by which strains are transmitted from environments and animals to food and the food production environment (FPE). In this context, we report, for the first time, the occurrence of a ST121 (CC121) strain isolated from a dolphin brain. The genome was compared with the genomes of 376 CC121 strains. Genomic comparisons showed that 16 strains isolated from food were the closest to the dolphin strain. Like most of the food strains analyzed here, the dolphin strain included genomic features (transposon Tn*6188*, plasmid pLM6179), both described as being associated with the strain’s adaptation to the FPE. Like all 376 strains, the dolphin strain contained a truncated *actA* gene and *inlA* gene, both described as being associated with attenuated virulence. Despite this fact, the strain was able to cross blood-brain barrier in immunosuppressed dolphin exposed polychlorinated biphenyl and invaded by parasites. Our data suggest that the dolphin was infected by a food-related strain released into the Mediterranean Sea.

## 1. Introduction

*Listeria monocytogenes* (*Lm*) is a facultative intracellular pathogen responsible for listeriosis, a serious food-borne disease affecting both humans and animals. *Lm* can infect diverse mammalian species leading to various symptoms: abortion, septicemia, gastroenteritis and central nervous system infections. *Lm* is a ubiquitous bacterium found in a variety of ecological niches [1,2]. There is increasing information on the prevalence of *Lm* in wild life, particularly in wild mammals. The presence of *Lm* in species of wild mammals such as rats, other rodents, hedgehogs, moose, otter, raccoon, deers, fallow deers, wild boars, bears, foxes and monkeys has been reported several times [3,4,5,6,7,8,9,10]. These studies indicate that these species can act as a transmission vehicle and play an ecological role as a reservoir of *Lm*. Regarding aquatic mammals, to our knowledge, only one study reported the presence of *Lm* isolated from a dolphin [11].

*Lm* is a genetically heterogeneous species divided into four phylogenetic lineages, of which lineages I and II are the most frequently encountered. Lineage I strains are overrepresented in human clinical infections and in ruminant neurolisteriosis. Lineage II strains are common in food, widespread in the natural and farm environments, and are also commonly isolated from animal listeriosis and sporadic human clinical cases [12]. MLST-Clonal complexes (CCs) and sequences types (STs) are systematically used to classify strain populations [13,14,15,16]. ST is defined as the unique association of alleles from the seven housekeeping genes and CC as a cluster of STs sharing at least six alleles out of 7. In lineage II, clonal complex (CC121) is a frequently reported genotype belonging to the few clones known to be epidemiologically important in Europe and worldwide [15,17]. CC121 strains pose a serious challenge to the food industry, as they are overrepresented in all food sectors [18,19], and can persist in different food processing plants for years [20,21,22,23,24,25]. Remarkably, in farming animals, CC121 strains are either never observed [26,27,28,29] or observed with a low prevalence [16,30]. Regarding wild mammals, only one study has reported the occurrence of CC121 strains among hedgehogs, in the UK [4].

A low incidence of human CC121 strains has been described during recent years [16,17,31,32,33]. Compared to CC1, CC4 and CC6 strains, CC121 strains have been shown to be hypovirulent in humanized mouse models and have been less often isolated in highly immuno-compromised patients [33]. CC121 strains are known to carry a truncated version of the Actine A (*actA*) and Internalin A (*inlA*) genes, both described as associated with attenuated virulence [22,34].

As little was previously known on how CC121 strains become part the food chain, we investigated their routes of transmission from outdoor environments and animals to food. This was one of the objectives of the European research project LISTADAPT (“Adaptive traits of *Listeria monocytogenes* to its diverse ecological niches”; https://onehealthejp.eu/jrp-listadapt/), which is part of the H2020 “One Health” European Joint Programme. The LISTADAPT consortium set up a broad-based, diverse and unique European collection of fully sequenced strains [27], collecting isolates from the natural environment, animals and foodstuff. It is in this context that we report the first identification of a ST121 strain isolated from a dolphin brain. The genome was compared with the genomes of a large dataset of 376 ST121 strains from different origins isolated in 25 European countries and USA. 

## 2. Results

### 2.1. Case Presentation

The subject was a female dolphin with severe hemorrhagic gastroenteritis, mild hepatomegaly, a hyperemic uterus and meninges and a severe infestation of the bronchial mucosa. Gross examination revealed hyperemia of the meninges, while histopathology identified widespread neuronal degeneration of the brain parenchyma, accompanied by satellitosis and neuronophagia. Moreover, multifocal mild vasculitis (infiltration by lymphoplasma cells) was detected around the vessels, associated with acute intracerebral hemorrhages. The lesions were all compatible with listeriosis. In the fat tissue, 30 ppm of Polychlorinatedbiphenyl (PCB) were found. Poor nutrition state and parasites were found such as cists of *Phillobotrium spp*. in the skin, *Pholeter gastrophilus* in the stomach, cysts of *Monorigma grimaldi* in the peritoneal cavity, and *Halocercus spp.* in the lung and bronchi. The bacterium *Photobacterium damselae* was also detected in lung and mediastinal lymph nodes.

### 2.2. Strain Characterization

The strain was characterized as belonging to serogroup IIa and sequence type ST121 through the ARtWORK pipeline.

### 2.3. Genomic Comparison

The genome of the strain isolated from dolphin (IT-OTH-CP-36) was compared with the genomes of 376 CC121 strains, 371 of which were isolated from 25 European countries and five from the USA bovine strains. A total of 318 strains were isolated from ready to eat (RTE) food (n = 318) (fish and fishery products (n = 174), meat and meat products (n = 81), dairy products (n = 6), vegetables/fruits (n = 22), composite dishes (n = 35)). The panel also included isolates from food processing environment (FPE) (n = 32), sporadic clinical cases (n = 3), farm animals (poultry, cattle and pigs) (n = 12), wild animals (wild boar and a hedgehog) (n = 3) and the natural or farm environment (n = 8). Of the 376 genomes, 232 were available in public databases, 107 were sequenced during the LISTADAPT project [27] and 37 genomes were sequenced specifically in the framework of this study.

#### 2.3.1. Phylogenomic Analysis

Most of the CC121 strains (367 out of 377) belonged to ST121, while one belonged to ST176, seven to ST326, one to ST593 and one to ST741.

**Figure 1:**
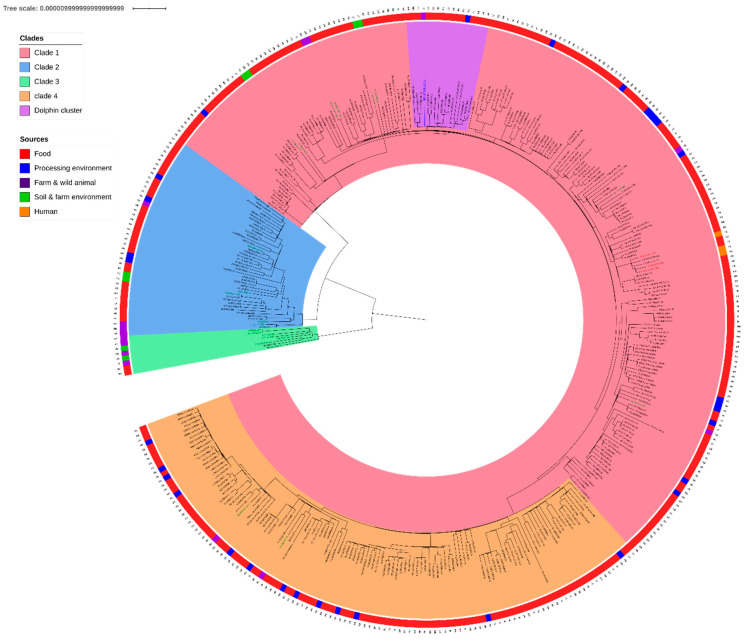
Recombination-aware phylogeny of CC121 (377 strains). CC121 has four distinct clades (clade 1 in red, clade 2 in blue clade 3 in green and clade 4 in orange). The environmental and animal strains are in green. The so-called “dolphin cluster” containing strain IT-OTH-CP-36 (labelled in blue) isolated from the dolphin is in purple. The outer ring represents the origins of the strains.

Figure 1 presents the SNP based core genome phylogeny of the CC121. IT-OTH-CP-36 belongs to a large clade (n = 207) based on hierarchical clustering. Defined as clade 1, this clade includes the ten strains that does not belong to ST121 and eight out of 23 of the wild/farm animals and environment strains as well as the three human strains, 14 out of 32 strains isolated from the FPE and 182 out of the 318 food strains. The average pairwise distance of IT-OTH-CP-36 from the strains in this clade was 52 +/− 13 SNPs.

CC121 phylogenomic reconstruction shows three other clades. Clade 2 clusters 42 ST121 strains, including 31 food strains, two animal strain isolated from wild boar in Germany, two environmental strains, of which one was isolated from a strawberry field and the other from sewage water, both in the Czech Republic, three strains isolated from farm animals in France and Germany and four FPE strains. Clade 2 had a genomic distance of 191 +/− 52 SNPs from the others clades (52 +/− 18 SNPs inside this clade). 

Clade 3 clusters eight ST121 strains, including the five strains from the USA. Those strains were isolated from soil or farm animals and two were from food, one from cheese in Slovakia and the other from frozen berries in France. This clade had a distance of 1295 +/− 8 SNPs from the two other clades (54 +/− 26 SNPs inside this clade).

Clade 4 clusters 120 strains, including two animal strains (NL-AVI-UN-36 from poultry and BE-BOV-SK-H-71 from bovine), 13 FPE strains and 103 RTE strains. This clade is closely related to clade 1 (61 +/− 13 SNPs on average between those two clades, compared to 38 +/− 11 SNPs inside the clade 4).

The 17 animal and environmental strains from clades 1, 2 and 4 did not form a specific cluster, with the exception of duplicate strains HR-BOV-CP-I-46 and HR-BOV-CP-I-47 in clade 1 and duplicate strains 16SEL1187LM and 16SEL1188LM in clade 2. 

The dolphin strain belongs to a cluster entitled “dolphin cluster” (Figure 1, in purple) including 16 food strains (pairwise distance of 26 +/− 10 SNPs) that were mainly isolated from fish and meat between 2005 and 2015 in six different countries (Denmark (n = 2), France (n = 7), Hungary (n = 2), Italy (n = 1), Spain (n = 3) and Sweden (n = 1)). The dolphin strain was not linked to any other animal or environmental strains. On average, the dolphin strain had a pairwise distance of 55 +/− 12 SNPs from the animal and environmental strains in clade 1. The closest strain (UK-OTH-FE-U-5) differed by 41 SNPs.

#### 2.3.2. Investigation of Factors Associated with Virulence

Table 1 summarizes the virulence genes detected in this study. Like all the 376 strains analyzed here, the dolphin strain harbored (i) the listeria Stress Survival Islet 2 (SSI2) containing the genes *lin0464* and *lin0465*; (ii) the truncated *inlA* gene; (iii) a deletion of 105 bp inside the *actA* gene; (iv) a gap of six nucleotides in the *inlK* gene; (v) a gap of 18 nucleotides in the *cwhA* gene.

#### 2.3.3. Pan-Genome Investigation

Pan-genome analysis revealed a core genome of 2525 genes with 111 soft-core genes (i.e. in more than 95% of the genomes). The pan-genome of the 377 CC121 strains included 691 genes, present in 15% to 95% of the genomes, alongside 2523 genes present in less than 15% of the genomes.

**Figure 2:**
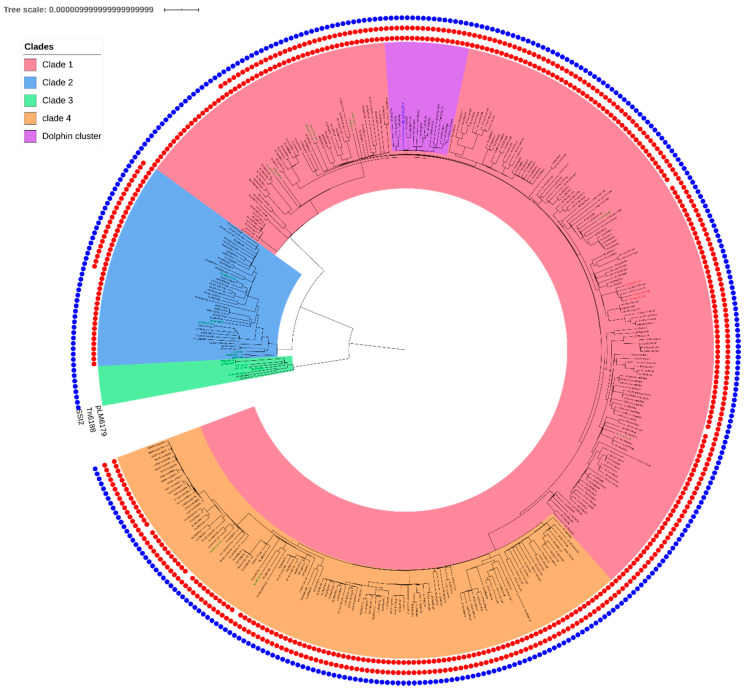
Presence of mobile elements and SSI2 in CC121 (377 strains) in regards of the phylogeny. The inner red dots correspond to pLM6179. The second red dots circle corresponds to Tn*6188* and the blue circle to SSI2.

Genome wide association studies (GWAS) revealed that, like the strains of clades 1 and 2, the dolphin strain was characterized by the presence of (i) plasmid, pLM6179, with 99% of conservation in clade 1 and 98% in clade 2 and (ii) transposon, Tn*6188*, conserved of respectively 94% and 55% in clades 1 and 2. There were very few genomic differences between clades 1 and 2, except for the presence of two genes (group_3191 coding for a hypothetical protein and the secreted effector protein *sseB*) observed only in clade 1 (associated Bonferroni adjusted *p*-value < 1.00 × 10^−33^). Clade 3 was characterized by the absence of plasmid, pLM6179, and transposon Tn*6188* and the presence of a cluster of two genes: a glycoxalase-like protein and hypothetical protein, not observed in clades 1 and 2 (adjusted *p* value = 1.02 × 10^−13^). Figure 2 presents the mobile elements present in every genome.

According to pan genome analysis, the dolphin strain presented a single uncommon (14/377) gene corresponding to an Insertion Sequence 3 (IS3) family transposase. According to association studies, the dolphin cluster presented a unique associated genomic feature: an insertion of two Helix Turn Helix (HTH) type transcriptional regulators (adjusted *p*-value = 1.06 × 10^−10^). This insertion was present in 16 out of 17 genomes. It was, however, absent from the strain IT-OTH-CP-36.

## 3. Discussion

Although increasing reports are available on the prevalence of *Lm* in diverse wild species [9,16,29,35,36], it is rare to isolate *Lm* strains from a dolphin. In England, systematic surveillance programs were conducted on 728 cetaceans of 16 different species tested for 12 consecutive years and no *Lm* strain was ever detected [37]. Only one study in 2015 reported *Lm* infection in a dolphin along the coast of Liguria in Italy. The strain was subtyped as ST399 and was linked to a coinfection with *Toxoplasma gondii* and *Brucella* [11]. Our study is, therefore, the first to report the occurrence in a cetacean of a strain of *Lm* belonging to ST121. 

Most of the 376 CC121 genomes analyzed in this study (320) were of strains isolated from food and the FPE. This was consistent with the fact that CC121 is a food-associated clonal complex [33]. The dolphin strain was part of the largest CC121 clade that includes most of the food and food processing environment strains. It is notable that this strain belonged to a cluster including only food strains isolated, from various food sectors, in six different European countries. In addition, like the ST121 food strains, the dolphin strain harbored the Tn*6188* transposon carrying the *qacC* resistance gene, which confers increased tolerance towards various quaternary ammonium compounds [38,39,40] and plasmid pLM6179, carrying the *cadAC* resistance gene, which contributes to tolerance against elevated temperature, salinity, acidic environments, oxidative stresses and disinfectants [22,37,41]. The presence of these key genomic factors contributes to the success of ST121 strains in food production [19,22,33,42]. In our study, these factors were not harbored by the strains in clade 3, which is notably associated with animal and environmental strains. 

The dolphin strain did not have any of the genetic features that would indicate higher virulence than the other CC121 strains. We investigated several key virulence genes. The dolphin strain had the same deletions as those observed in the genomes of the 376 CC121 strains. It contained a truncated *inlA* gene (insertion of a stop codon), known to be the main factor for invading intestinal epithelial cells [22,34] and a deletion of 105 pb in *actA,* a gene involved in cell to cell mobility and biofilm formation [22,33]. There was a gap in the *inlK* gene, known to encode an immune modulator. This protein is involved in autophagy evasion and plays a critical role in *Lm* virulence [43]. The dolphin strain also had a gap in the *cwhA* gene (cell wall hydrolase A). This gene is known to encode for the p60 protein, a major virulence factor involved in intra- and intercellular spread of *Lm* [44]. 

Exposure to anthropogenic contaminants, such as polychlorinatedbiphenyl (PCB), is known to have an immunosuppressive effect on cetaceans [45,46,47,48]. In this study, the dose of 30 ppm in fat tissues was two times higher than the threshold levels accepted, i.e., a 17 ppm lipid base for PCBs [47,49,50,51]. Such exposure may explain the dolphin’s susceptibility to *Lm*. Moreover, this susceptibility could also be linked to a co-infection with the many parasites found in the dolphin’s brain. 

Recent studies compared the genomic background of the CC121 strains between food, food processing environments and sporadic human cases [41,52] [22,42]. For instance, Harter et al. [42] recently identified a stress survival islet 2 (SSI-2) in ST121 strains that confer resistance to alkaline and oxidative stress and improved adaptation to conditions routinely encountered in the food processing environments. SSI-2 was predominantly but not exclusively present in the 476 ST121 isolates from human cases and from food, analyzed by Harter et al. In our study, we showed that SSI-2 was (i) highly conserved among the strain panel and (ii) present not only in the 351 food and three human cases strains analyzed here, but also the 14 animal and the eight environmental strains. Our study is the first to get insight within the genetic make-up of ST121 isolates from natural/farm environment and wild/farm animals and contributed to improve our knowledge on the genetic population structure of ST121 strains.

The presence of *Lm* in marine waters has been described in the literature [53,54,55]. Although *Lm* is not considered a marine micro-organism, it can survive for about 3 weeks in sea water [56]. The presence of *Lm* in coastal water is common [57] and often correlated with urban activities along the coastline [58]. Additionally, *Lm* is known to be present in water treatment facilities [48]. The Mediterranean Sea has a slow renewal rate (of 125 years) [59] and has a dense population. This results in an accumulation of anthropic pollution such as wastewater and detritus and consequently, the exposure of marine animals to pathogens, linked to human activity. It is likely that *Lm* food strains have spread to the coastal environment. Given the data we obtained, it is likely that the infection of a dolphin by *Lm* may be the result of environmental contamination by anthropogenic activities. The infection of this dolphin by a strain of a clonal complex known to be hypovirulent for human asks the question of the degree of weakness of the animal and its degree of exposure to this clone. This study highlights the necessity (i) to assess the dissemination of potential pathogens through human waste into marine environment; and (ii) to improve monitoring of wild animal health.

## 4. Materials and Methods

### 4.1. Case Presentation

In February 2017, a listeriosis case was reported in Italy, which was detected in the brain of a wild dolphin stranded on a beach near Orbetello in Tuscany, northwestern Italy. Necropsy was performed according to the protocol of the National Reference Center for Diagnostic Procedures in Stranded Mammals (Centro di Referenza Nazionale per le Indagini Diagnostiche sui Mammiferi marini spiaggiati, C.Re.Di.Ma, DL 15A00179, 22 October 2014). 

### 4.2. Detection of Lm

*Lm* was sought during analyses carried out at the Istituto Zooprofilattico Sperimentale del Lazio e della Toscana whose detection protocol for stranded marine mammals includes testing for Lm in compliance with Chapter 2.9.7 of the OIE Manual of Diagnostic Tests and Vaccines for Terrestrial Animals- Listeria monocytogenes [60]. The Lm strain detected, named “IT-OTH-CP-36”, was isolated on sheep blood agar and sent to Istituto Zooprofilattico Sperimentale dell’Abruzzo e Molise G.Caporale (ISZAM) as a pure culture for sequencing.

### 4.3. DNA Extraction and Whole Genome Sequencing 

DNA was extracted from the IT-OTH-CP-36 strain using the Maxwell® 16 Cell DNA Purification Kit, according to the manufacturer’s instructions (Promega, Madison, WI, USA) then used for library preparation using the Nextera XT library preparation kit. Sequencing was performed on the NextSeq 500 platform. NGS based MLST prediction determined the ST and the CC.

The IT-OTH-CP-36 genome was compared to all the CC121 genomes available from the ANSES Laboratory for Food Safety database, including 107 genomes described by Félix et al. [27], 54 genomes described by Henri et al. [18], 6 by Fritsch et al. [61], 29 by Palma et al. [25], 143 genomes from the Liseq collection [17] and 21 new genomes specifically sequenced in the framework of this study. These genomes included 11 genomes of strains from Ireland, four from Republic of North Macedonia, four from France and two from Czech Republic. 

To provide a positive control under the hypothesis of a differentiation of the environmental and RTE strains of CC121, two known duplicates described by Félix et al. [27] (HR-BOV-CP-I-46 and HR-BOV-CP-I-47) were included in this study. Additionally, five genomes of farm animals strains from USA, FSL F3-194, FSL F3-0293, FSL F3-146 [62], and the strains TB0512 (accession n°SRR6116310) and TB0511 (accession n°SRR6116326) [63] were included as comparison point and as an outgroup for phylogeny construction. The supplementary Table S1 describes the collection and details the quality of the 377 genomes included in this study.

The reads of the whole collection were processed, assembled and annotated through a harmonized in-house workflow called ARTwork (Assembly of Reads and Typing workflow) implemented in the ANSES Laboratory for Food Safety. This tool has been described in details by Villa nova et al. [64] and is available at: https://github.com/afelten-Anses/ARtWORK.

Normalized reads (i.e. 100x), scaffold assembly and annotated genomes were stored in an in house genomic database in the ANSES Laboratory for Food Safety. Sequences with a mean coverage bellow 30× were excluded (n = 2), as well as sequences with an abnormal assembly length (n = 2) or poor assembly quality (i.e. >200 contigs). In addition, inter-species and intra-species cross contaminations were evaluated using the confindR software (V. 0.5.1) [65]. The genomes that were predicted to be contaminated (n = 18) were excluded and the final dataset was of 377 genomes, including the strain isolated from the dolphin. The metadata associated with the strain collection used in this study are detailed in Supplementary Table S1.

### 4.4. Phylogenomic Reconstruction of the CC121 Strain Population Structure

A phylogenetic tree was produced using the iVARcall2 pipeline [66] to align the reads on a reference (LM6179 accession number: HG813249.1) and to determine pairwise single nucleotide substitution (SNP) distances. The phylogenetic tree was calculated on the pseudogenome produced by the iVARcall2 workflow selecting the best model using IQ-TREE V.1.6.9 [67]. The best-fitted model for this dataset was determined as a three-substitution type model with equal base parameter, empirical base frequencies [68] and an allowed proportion of invariant sites (K3P+F+I). The tree was corrected for homologous recombination events using ClonalframeML [69] and visualized using iTOL [70]. Nested population structure (i.e. clades) were determined by hierarchical clustering using the RhierBAPS R package [71,72].

### 4.5. Identification of Virulence Factors and Genome Wide Association Study

Virulence factors were identified with the ABRicate software (V.0.8.10) (https://github.com/tseemann/abricate) using the VFDB database [73]. The plasmids were predicted using the MOB-suite tool (V. 1.4.9). Pan-genome content was identified through annotated GFF files produced by Prokka [74] and ARTwork processing of the reads then analyzed using Panaroo (V. 1. 2.2) [75]. Specific traits associations were then tested using Scoary software (V. 1.6.16) [76] to identify (i) the genomic features associated with the different CC121 clades and (ii) specifics markers of the dolphin cluster. 

## Figures and Tables

**Table 1:** Virulence factors identified in a significant proportion of CC121 strains. The alleles are based on the strain IT-OTH-CP-36, which presented, in each case, the majoritarian allele. The number of strains presenting this allele is indicated in the “n consensus” column.

Gene	Coverage	Coverage_Map	Gaps	n Consensus	%Identity	%Coverage	Accession	Product
*inlJ*	1-1934/2556	========/===...	#NA	319	95.56	75.55	NP_466343	(inlJ) internalin J
*inlJ*	1589-2556/2556	.........======	#NA	286	90.39	37.87	NP_466343	(inlJ) internalin J
*prfA*	1-714/714	===============	0/0	377	99.86	100.00	NP_463731	(prfA) listeriolysin positive regulatory protein
*plcA*	1-954/954	===============	0/0	377	98.64	100.00	NP_463732	(plcA) phosphatidylinositol-specific phospholipase c
*hly*	1-1590/1590	===============	0/0	371	99.69	100.00	NP_463733	(hly) listeriolysin O precursor
*mpl*	1-1533/1533	===============	0/0	374	99.61	100.00	NP_463734	(mpl) Zinc metalloproteinase precursor
*actA*	1-923/1920	========.......	0/0	376	97.94	48.07	NP_463735	(actA) actin-assembly inducing protein precursor
*actA*	895-1920/1920	......=========	0/0	374	96.78	53.44	NP_463735	(actA) actin-assembly inducing protein precursor
*plcB*	1-870/870	===============	0/0	374	99.54	100.00	NP_463736	(plcB) phospholipase C
*clpC*	1-2463/2463	===============	0/0	358	99.80	100.00	NP_463763	(clpC) endopeptidase Clp ATP-binding chain C
*vip*	1-1200/1200	===============	0/0	373	99.42	100.00	NP_463850	(vip) surface adhesin Vip
*pdgA*	1-1401/1401	===============	0/0	375	99.93	100.00	NP_463944	(pdgA) Peptidoglycan N-deacetylase
*inlA*	1-2403/2403	===============	0/0	360	97.79	100.00	NP_463962	(inlA) Internalin A
*inlB*	1-1893/1893	===============	0/0	367	99.10	100.00	NP_463963	(inlB) Internalin B
*iap/cwhA*	1-1449/1449	========/======	1/18	374	97.45	98.76	NP_464110	(iap/cwhA) P60 extracellular protein invasion associated protein Iap
*hpt*	1-1386/1386	===============	0/0	366	97.91	100.00	NP_464364	(hpt) hexose phosphate transport protein
*lplA1*	1-996/996	===============	0/0	370	98.49	100.00	NP_464456	(lplA1) lipoate protein ligase
*clpE*	1-2175/2175	===============	0/0	361	99.86	100.00	NP_464522	(clpE) ATP-dependent protease
*aut*	1-1719/1719	===============	0/0	360	99.83	100.00	NP_464601	(aut) autolysin
*ami*	1-2754/2754	===============	0/0	292	99.53	100.00	NP_466081	(ami) autolysin amidase adhesin
*gtcA*	1-438/438	===============	0/0	375	100	100.00	NP_466072	(gtcA) wall teichoic acid glycosylation protein GtcA
*clpP*	1-597/597	===============	0/0	358	100	100.00	NP_465991	(clpP) ATP-dependent Clp protease proteolytic subunit
*inlK*	1-1797/1797	========/======	1/6	322	98.50	100.00	NP_464815	(inlK) internalin K
*oatA*	1-1869/1869	===============	0/0	367	99.62	100.00	NP_464816	(oatA) peptidoglycan O-acetyltransferase
*lap*	1-2601/2601	===============	0/0	324	99.92	100.00	NP_465159	(lap) Listeria adhesion protein Lap
*lapB*	1-5136/5136	===============	0/0	329	99.10	100.00	NP_465191	(lapB) Listeria adhesion protein LapB
*inlC*	1-891/891	===============	0/0	362	99.89	100.00	NP_465311	(inlC) internalin C
*fbpA*	1-1713/1713	===============	0/0	311	99.83	100.00	NP_465354	(fbpA) fibronectin-binding protein
*lspA*	1-465/465	===============	0/0	372	100	100.00	NP_465369	(lspA) signal peptidase II
*lpeA*	1-933/933	===============	0/0	369	99.79	100.00	NP_465372	(lpeA) lipoprotein promoting cell invasion
*bsh*	1-978/978	===============	0/0	369	100	100.00	NP_465591	(bsh) bile salt hydrolase
*prsA2*	1-882/882	===============	0/0	375	99.89	100.00	NP_465743	(prsA2) post translocation chaperone PrsA2

## Supplementary Materials

The following are available online at https://www.mdpi.com/2076-0817/9/10/802/s1, Table S1: strains metadata, genome assembly quality and reads accession of the CC121 collection.

## References

[1] Piveteau P., Depret G., Pivato B., Garmyn D., Hartmann A. (2011). Changes in Gene Expression during Adaptation of Listeria monocytogenes to the Soil Environment. PLoS ONE.

[2] Vivant A.L., Garmyn D., Piveteau P. (2013). Listeria monocytogenes, a down-to-earth pathogen. Front. Cell. Infect. Microbiol..

[3] Giovannini A., Cancellotti F.M., Turilli C., Randi E. (1988). Serological investigations for some bacterial and viral pathogens in fallow deer (Cervus dama) and wild boar (Sus scrofa) of the San Rossore Preserve, Tuscany, Italy. J. Wildl. Dis..

[4] Hydeskov H.B., Amar C.F.L., Fernandez J.R., John S.K., Macgregor S.K., Cunningham A.A., Lawson B. (2019). Listeria Monocytogenes Infection of Free-Living Western European Hedgehogs (Erinaceus Europaeus). J. Zoo Wildl. Med..

[5] Inoue S., Iida T., Tanikawa T., Maruyama T., Morita C. (1991). Isolation of Listeria monocytogenes from roof rats (Rattus rattus) in buildings in Tokyo. J. Vet. Med. Sci..

[6] Inoue S., Tanikawa T., Kawaguchi J., Iida T., Morita C. (1992). Prevalence of Listeria (spp.) in wild rats captured in the Kanto area of Japan. J. Vet. Med. Sci..

[7] Lyautey E., Lapen D.R., Wilkes G., McCleary K., Pagotto F., Tyler K., Hartmann A., Piveteau P., Rieu A., Robertson W.J. (2007). Distribution and Characteristics of *Listeria monocytogenes* Isolates from Surface Waters of the South Nation River Watershed, Ontario, Canada. Appl. Environ. Microbiol..

[8] Parsons C., Niedermeyer J., Gould N., Brown P., Strules J., Parsons A.W., Bernardo Mesa-Cruz J., Kelly M.J., Hooker M.J., Chamberlain M.J. (2019). Listeria monocytogenes at the human–wildlife interface: Black bears (Ursus americanus) as potential vehicles for Listeria. Microb. Biotechnol..

[9] Weindl L., Frank E., Ullrich U., Heurich M., Kleta S., Ellerbroek L., Gareis M. (2016). Listeria monocytogenes in Different Specimens from Healthy Red Deer and Wild Boars. Foodborne Pathog. Dis..

[10] Yoshida T., Sugimoto T., Sato M., Hirai K. (2000). Incidence of Listeria monocytogenes in wild animals in Japan. J. Vet. Med. Sci..

[11] Grattarola C., Giorda F., Iulini B., Pintore M.D., Pautasso A., Zoppi S., Goria M., Romano A., Peletto S., Varello K. (2016). Meningoencephalitis and Listeria monocytogenes, Toxoplasma gondii and Brucella spp. coinfection in a dolphin in Italy. Dis. Aquat. Org..

[12] Orsi R.H., Bakker H.C., Wiedmann M. (2011). Listeria monocytogenes lineages: Genomics, evolution, ecology, and phenotypic characteristics. Int. J. Med. Microbiol..

[13] Ragon M., Wirth T., Hollandt F., Lavenir R., Lecuit M., Le Monnier A., Brisse S. (2008). A new perspective on Listeria monocytogenes evolution. PLoS Pathog..

[14] Chenal-Francisque V., Lopez J., Cantinelli T., Caro V., Tran C., Leclercq A., Lecuit M., Brisse S. (2011). Worldwide distribution of major clones of Listeria monocytogenes. Emerg. Infect. Dis..

[15] Cantinelli T., Chenal-Francisque V., Diancourt L., Frezal L., Leclercq A., Wirth T., Lecuit M., Brisse S. (2013). "Epidemic clones" of Listeria monocytogenes are widespread and ancient clonal groups. J. Clin. Microbiol..

[16] Haase J.K., Didelot X., Lecuit M., Korkeala H., Achtman M. (2014). The ubiquitous nature of Listeria monocytogenes clones: A large-scale Multilocus Sequence Typing study. Environ. Microbiol..

[17] Painset A., Björkman J.T., Kiil K., Guillier L., Mariet J.-F., Félix B., Amar C., Rotariu O., Roussel S., Perez-Reche F. (2019). LiSEQ—Whole-genome sequencing of a cross-sectional survey of Listeria monocytogenes in ready-to-eat foods and human clinical cases in Europe. Microb. Genom..

[18] Henri C., Felix B., Guillier L., Leekitcharoenphon P., Michelon D., Mariet J.F., Aarestrup F.M., Mistou M.Y., Hendriksen R.S., Roussel S. (2016). Population Genetic Structure of Listeria monocytogenes Strains as Determined by Pulsed-Field Gel Electrophoresis and Multilocus Sequence Typing. Appl. Environ. Microbiol..

[19] Felix B., Feurer C., Maillet A., Guillier L., Boscher E., Kerouanton A., Denis M., Roussel S. (2018). Population Genetic Structure of *Listeria monocytogenes* Strains Isolated From the Pig and Pork Production Chain in France. Front. Microbiol..

[20] Holch A., Webb K., Lukjancenko O., Ussery D., Rosenthal B.M., Gram L. (2013). Genome sequencing identifies two nearly unchanged strains of persistent *Listeria monocytogenes* isolated at two different fish processing plants sampled 6 years apart. Appl. Environ. Microbiol..

[21] Ortiz S., Lopez V., Martinez-Suarez J.V. (2014). Control of *Listeria monocytogenes* contamination in an Iberian pork processing plant and selection of benzalkonium chloride-resistant strains. Food Microbiol..

[22] Rychli K., Wagner E.M., Ciolacu L., Zaiser A., Tasara T., Wagner M., Schmitz-Esser S. (2017). Comparative genomics of human and non-human *Listeria monocytogenes* sequence type 121 strains. PLoS ONE.

[23] Pasquali F., Palma F., Guillier L., Lucchi A., De Cesare A., Manfreda G. (2018). *Listeria monocytogenes* Sequence Types 121 and 14 Repeatedly Isolated Within One Year of Sampling in a Rabbit Meat Processing Plant: Persistence and Ecophysiology. Front. Microbiol..

[24] Stoller A., Stevens M.J.A., Stephan R., Guldimann C. (2019). Characteristics of *Listeria monocytogenes* Strains Persisting in a Meat Processing Facility over a 4-Year Period. Pathogens.

[25] Palma F., Brauge T., Radomski N., Mallet L., Felten A., Mistou M.-Y., Brisabois A., Guillier L., Midelet-Bourdin G. (2020). Dynamics of mobile genetic elements of *Listeria monocytogenes* persisting in ready-to-eat seafood processing plants in France. BMC Genom..

[26] Dreyer M., Aguilar-Bultet L., Rupp S., Guldimann C., Stephan R., Schock A., Otter A., Schupbach G., Brisse S., Lecuit M. (2016). *Listeria monocytogenes* sequence type 1 is predominant in ruminant rhombencephalitis. Sci. Rep..

[27] Félix B., Sevellec Y., Palma F., Felten A., Radomski N., Mallet L., Blanchard Y., Leroux A., Soumet C., Bridier A. (2020). A European-wide dataset to decipher adaptation mechanisms of *Listeria monocytogenes* to diverse ecological niches (unpublished, in revision). Scientific Data.

[28] Linke K., Rückerl I., Brugger K., Karpiskova R., Walland J., Muri-Klinger S., Tichy A., Wagner M., Stessl B. (2014). Reservoirs of listeria species in three environmental ecosystems. Appl. Environ. Microbiol..

[29] Papić B., Pate M., Félix B., Kušar D. (2019). Genetic diversity of *Listeria monocytogenes* strains in ruminant abortion and rhombencephalitis cases in comparison with the natural environment. BMC Microbiol..

[30] Steckler A.J., Cardenas-Alvarez M.X., Townsend Ramsett M.K., Dyer N., Bergholz T.M. (2018). Genetic characterization of *Listeria monocytogenes* from ruminant listeriosis from different geographical regions in the U.S. Vet. Microbiol..

[31] Althaus D., Lehner A., Brisse S., Maury M., Tasara T., Stephan R. (2014). Characterization of *Listeria monocytogenes* strains isolated during 2011-2013 from human infections in Switzerland. Foodborne Pathog. Dis..

[32] Kwong J.C., Mercoulia K., Tomita T., Easton M., Li H.Y., Bulach D.M., Stinear T.P., Seemann T., Howden B.P. (2016). Prospective Whole-Genome Sequencing Enhances National Surveillance of *Listeria monocytogenes*. J. Clin. Microbiol..

[33] Maury M.M., Tsai Y.H., Charlier C., Touchon M., Chenal-Francisque V., Leclercq A., Criscuolo A., Gaultier C., Roussel S., Brisabois A. (2016). Uncovering *Listeria monocytogenes* hypervirulence by harnessing its biodiversity. Nat. Genet..

[34] Rychli K., Stessl B., Szakmary-Brändle K., Strauß A., Wagner M., Schoder D. (2018). *Listeria monocytogenes* Isolated from Illegally Imported Food Products into the European Union Harbor Different Virulence Factor Variants. Genes.

[35] Hellstrom S., Kiviniemi K., Autio T., Korkeala H. (2008). *Listeria monocytogenes* is common in wild birds in Helsinki region and genotypes are frequently similar with those found along the food chain. J. Appl. Microbiol..

[36] Gismervik K., Aspholm M., Rorvik L.M., Bruheim T., Andersen A., Skaar I. (2015). Invading slugs (Arion vulgaris) can be vectors for *Listeria monocytogenes*. J. Appl. Microbiol..

[37] Naditz A.L., Dzieciol M., Wagner M., Schmitz-Esser S. (2019). Plasmids contribute to food processing environment–associated stress survival in three *Listeria monocytogenes* ST121, ST8, and ST5 strains. Int. J. Food Microbiol..

[38] Müller A., Rychli K., Muhterem-Uyar M., Zaiser A., Stessl B., Guinane C.M., Cotter P.D., Wagner M., Schmitz-Esser S. (2013). Tn6188—A novel transposon in *Listeria monocytogenes* responsible for tolerance to benzalkonium chloride. PLoS ONE.

[39] Müller A., Rychli K., Zaiser A., Wieser C., Wagner M., Schmitz-Esser S. (2014). The *Listeria monocytogenes* transposon Tn6188 provides increased tolerance to various quaternary ammonium compounds and ethidium bromide. FEMS Microbiol. Lett..

[40] Ortiz S., López-Alonso V., Rodríguez P., Martínez-Suárez J.V. (2016). The Connection between Persistent, Disinfectant-Resistant *Listeria monocytogenes* Strains from Two Geographically Separate Iberian Pork Processing Plants: Evidence from Comparative Genome Analysis. Appl. Environ. Microbiol..

[41] Schmitz-Esser S., Muller A., Stessl B., Wagner M. (2015). Genomes of sequence type 121 *Listeria monocytogenes* strains harbor highly conserved plasmids and prophages. Front. Microbiol..

[42] Harter E., Wagner E.M., Zaiser A., Halecker S., Wagner M., Rychli K. (2017). Stress Survival Islet 2, Predominantly Present in *Listeria monocytogenes* Strains of Sequence Type 121, Is Involved in the Alkaline and Oxidative Stress Responses. Appl. Environ. Microbiol..

[43] Dortet L., Mostowy S., Samba-Louaka A., Gouin E., Nahori M.A., Wiemer E.A., Dussurget O., Cossart P. (2011). Recruitment of the major vault protein by InlK: A *Listeria monocytogenes* strategy to avoid autophagy. PLoS Pathog..

[44] Pilgrim S., Kolb-Maurer A., Gentschev I., Goebel W., Kuhn M. (2003). Deletion of the gene encoding p60 in *Listeria monocytogenes* leads to abnormal cell division and loss of actin-based motility. Infect. Immun..

[45] Cardellicchio N. (1995). Persistent contaminants in dolphins: An indication of chemical pollution in the mediterranean sea. Water Sci. Technol..

[46] Fossi M.C., Panti C., Marsili L., Maltese S., Spinsanti G., Casini S., Caliani I., Gaspari S., Muñoz-Arnanz J., Jimenez B. (2013). The Pelagos Sanctuary for Mediterranean marine mammals: Marine Protected Area (MPA) or marine polluted area? The case study of the striped dolphin (*Stenella coeruleoalba*). Mar. Pollut. Bull..

[47] Jepson P.D., Deaville R., Barber J.L., Aguilar À., Borrell A., Murphy S., Barry J., Brownlow A., Barnett J., Berrow S. (2016). PCB pollution continues to impact populations of orcas and other dolphins in European waters. Sci. Rep..

[48] Paillard D., Dubois V., Thiebaut R., Nathier F., Hoogland E., Caumette P., Quentin C. (2005). Occurrence of *Listeria* spp. in effluents of French urban wastewater treatment plants. Appl. Environ. Microbiol..

[49] Jepson P.D., Bennett P.M., Deaville R., Allchin C.R., Baker J.R., Law R.J. (2005). Relationships between polychlorinated biphenyls and health status in harbor porpoises (*Phocoena phocoena*) stranded in the United Kingdom. Environ. Toxicol. Chem..

[50] Kannan K., Blankenship A.L., Jones P.D., Giesy J.P. (2000). Toxicity Reference Values for the Toxic Effects of Polychlorinated Biphenyls to Aquatic Mammals. Hum. Ecol. Risk Assess..

[51] Marsili L., D’Agostino A., Bucalossi D., Malatesta T., Fossi M.C. (2004). Theoretical models to evaluate hazard due to organochlorine compounds (OCs) in Mediterranean striped dolphin (Stenella coeruleoalba). Chemosphere.

[52] Palma F., Pasquali F., Lucchi A., De Cesare A., Manfreda G. (2017). Whole genome sequencing for typing and characterisation of *Listeria monocytogenes* isolated in a rabbit meat processing plant. Ital. J. Food Saf..

[53] Beleneva I.A. (2011). Incidence and characteristics of *Staphylococcus aureus* and *Listeria monocytogenes* from the Japan and South China seas. Mar. Pollut. Bull..

[54] Bou-m’handi N., Jacquet C., El Marrakchi A., Martin P. (2007). Phenotypic and molecular characterization of *Listeria monocytogenes* strains isolated from a marine environment in Morocco. Foodborne Pathog. Dis..

[55] Zaytseva E., Ermolaeva S., Somov G.P. (2007). Low genetic diversity and epidemiological significance of *Listeria monocytogenes* isolated from wild animals in the far east of Russia. Infect. Genet. Evolut..

[56] Bremer P.J., Osborne C.M., Kemp R.A., Smith J.J. (1998). Survival of *Listeria monocytogenes* in sea water and effect of exposure on thermal resistance. J. Appl. Microbiol..

[57] Jami M., Ghanbari M., Zunabovic M., Domig K.J., Kneifel W. (2014). *Listeria monocytogenes* in Aquatic Food Products—A Review. Compr. Rev. Food Sci. Food Saf..

[58] Hutchison M., Thomas D., Strachan N., Goodburn K., Rotariu O. (2012). A review of the published literature and current production and processing practices in smoked fish processing plants with emphasis on contamination by Listeria monocytogenes. Final FSA Rep..

[59] Bergamasco A., Malanotte-Rizzoli P. (2010). The circulation of the Mediterranean Sea: A historical review of experimental investigations. Adv. Oceanogr. Limnol..

[60] OIE (2018). Chapter 2.9.7 *Listeria monocytogenes*. Manual of Diagnostic Tests and Vaccines for Terrestrial Animals 2019.

[61] Fritsch L., Felten A., Palma F., Mariet J.-F., Radomski N., Mistou M.-Y., Augustin J.-C., Guillier L. (2019). Insights from genome-wide approaches to identify variants associated to phenotypes at pan-genome scale: Application to *Listeria monocytogenes*’ ability to grow in cold conditions. Int. J. Food Microbiol..

[62] Fugett E.B., Schoonmaker-Bopp D., Dumas N.B., Corby J., Wiedmann M. (2007). Pulsed-field gel electrophoresis (PFGE) analysis of temporally matched *Listeria monocytogenes* isolates from human clinical cases, foods, ruminant farms, and urban and natural environments reveals source-associated as well as widely distributed PFGE types. J. Clin. Microbiol..

[63] Pirone-Davies C., Chen Y., Pightling A., Ryan G., Wang Y., Yao K., Hoffmann M., Allard M.W. (2018). Genes significantly associated with lineage II food isolates of *Listeria monocytogenes*. BMC Genom..

[64] Vila Nova M., Durimel K., La K., Felten A., Bessières P., Mistou M.-Y., Mariadassou M., Radomski N. (2019). Genetic and metabolic signatures of *Salmonella enterica* subsp. *enterica* associated with animal sources at the pangenomic scale. BMC Genom..

[65] Low A.J., Koziol A.G., Manninger P.A., Blais B., Carrillo C.D. (2019). ConFindr: Rapid detection of intraspecies and cross-species contamination in bacterial whole-genome sequence data. PeerJ.

[66] Felten A., Vila Nova M., Durimel K., Guillier L., Mistou M.-Y., Radomski N. (2017). First gene-ontology enrichment analysis based on bacterial coregenome variants: Insights into adaptations of *Salmonella* serovars to mammalian- and avian-hosts. BMC Microbiol..

[67] Schmidt H.A., Minh B.Q., von Haeseler A., Nguyen L.-T. (2014). IQ-TREE: A Fast and Effective Stochastic Algorithm for Estimating Maximum-Likelihood Phylogenies. Mol. Biol. Evol..

[68] Kimura M. (1981). Estimation of evolutionary distances between homologous nucleotide sequences. Proc. Natl. Acad. Sci. USA.

[69] Didelot X., Wilson D.J. (2015). ClonalFrameML: Efficient Inference of Recombination in Whole Bacterial Genomes. PLoS Comput. Biol..

[70] Letunic I., Bork P. (2019). Interactive Tree Of Life (iTOL) v4: Recent updates and new developments. Nucleic Acids Res..

[71] Cheng L., Connor T., Sirén J., Aanensen D., Corander J. (2013). Hierarchical and Spatially Explicit Clustering of DNA Sequences with BAPS Software. Mol. Biol. Evol..

[72] Tonkin-Hill G., Lees J., Bentley S., Frost S., Corander J. (2018). RhierBAPS: An R implementation of the population clustering algorithm hierBAPS. Wellcome Open Res..

[73] Liu B., Zheng D., Jin Q., Chen L., Yang J. (2018). VFDB 2019: A comparative pathogenomic platform with an interactive web interface. Nucleic Acids Res..

[74] Seemann T. (2014). Prokka: Rapid prokaryotic genome annotation. Bioinformatics.

[75] Tonkin-Hill G., MacAlasdair N., Ruis C., Weimann A., Horesh G., Lees J.A., Gladstone R.A., Lo S., Beaudoin C., Floto R.A. (2020). Producing Polished Prokaryotic Pangenomes with the Panaroo Pipeline. BioRxiv.

[76] Brynildsrud O., Bohlin J., Scheffer L., Eldholm V. (2016). Rapid scoring of genes in microbial pan-genome-wide association studies with Scoary. Genome Biol..

