# Supplementary material for: First Report on the Finding of Listeria mnocytogenes ST121 Strain in a Dolphin Brain
Source: Pathogens. 2020 Sep 28;9(10):802. doi: 10.3390/pathogens9100802 (PMC7601084; doi:10.3390/pathogens9100802)
Supplement: Supplementary file 1 [file pathogens-09-00802-s001.pdf]

Table S1. strains metadata, genome assembly quality and reads accession of the CC121 collection.

Genome_ID	Compartment	Details	Country	Year	ST	Reference	Contamstatus	Breadth coverage (%)	N50	Number of Contigs	Largest Contig	Total Assembly Length	Accession (Biosamble)
04CEB235LM	Fish and fishery products	smoked salmon	FR	2004	121	Henri et al. 2016	False	95,86	3042512	16	3042512	3112904	ERS1374952 (SAMEA4475773)
05CEB426LM	Fish and fishery products	salmon	FR	2005	121	Henri et al. 2016	False	94,55	3008570	25	3008570	3081975	ERS1374954 (SAMEA4475775)
05CEB745LM	Composite dishes	salmon sandwich	FR	2005	121	Henri et al. 2016	False	96,12	3003770	9	3003770	3066471	ERS1374964 (SAMEA4475785)
05CEB746LM	Composite dishes	pizza	FR	2005	121	Henri et al. 2016	False	95,82	3186668	11	3186668	3250531	ERS1374965 (SAMEA4475786)
06CEB377LM	Processing environment	surfaces	FR	2006	121	Henri et al. 2016	False	95,83	3033096	7	3033096	3059429	ERS1374983 (SAMEA4475804)
06CEB469LM	Composite dishes	ham and cheese sandwich	FR	2006	121	Henri et al. 2016	False	95,75	3185929	5	3185929	3248006	ERS1374987 (SAMEA4475808)
06CEB476LM	Composite dishes	pizza	FR	2006	121	Henri et al. 2016	False	96,13	3002921	5	3002921	3064685	ERS1374988 (SAMEA4475809)
07CEB276LM	Fish and fishery products	tarama	FR	2007	121	Henri et al. 2016	False	95,06	2883111	2	2883111	2944222	ERS1374993 (SAMEA4475814)
07CEB914LM	Meat and meat products	poultry meat	FR	2007	121	Henri et al. 2016	False	95,84	3001279	11	3001279	3064625	ERS1374997 (SAMEA4475818)
08CEB07LM	Processing environment	surfaces	FR	2008	121	Henri et al. 2016	False	95,79	2996799	8	2996799	3059301	ERS1374999 (SAMEA4475820)
08CEB107LM	Composite dishes	ham and emental sandwich	FR	2008	121	Henri et al. 2016	False	95,76	3057230	8	3057230	3059256	ERS1375001 (SAMEA4475822)
08CEB173LM	Meat and meat products	Sausage	FR	2008	121	Henri et al. 2016	False	95,99	3050503	24	3050503	3056893	ERS1375005 (SAMEA4475826)
08CEB196LM	Fish and fishery products	tarama	FR	2008	121	Henri et al. 2016	False	95,09	3035375	39	3035375	3112225	ERS1375008 (SAMEA4475829)
08CEB214LM	Composite dishes	rice and seafood	FR	2008	121	Henri et al. 2016	False	95,74	2995342	11	2995342	3058999	ERS1375010 (SAMEA4475831)

08CEB263LM	Processing environment	surfaces	FR	2008	121	Henri et al. 2016	False	95,02	3003458	50	3003458	3084532	ERS1375013 (SAMEA4475834)
08CEB279LM	Meat and meat products	merguez	FR	2008	121	Henri et al. 2016	False	95,18	3062244	3	3062244	3062894	ERS1375014 (SAMEA4475835)
08CEB44LM	Composite dishes	tortellini ricotta	FR	2008	121	Henri et al. 2016	False	95,28	3047336	12	3047336	3111304	ERS1375017 (SAMEA4475838)
08CEB473LM	Meat and meat products	ham	FR	2008	121	Henri et al. 2016	False	95,41	2987822	97	2987822	3081607	ERS1375018 (SAMEA4475839)
08CEB53LM	Composite dishes	sandwich jambon fromage	FR	2008	121	Henri et al. 2016	False	95,14	3061033	5	3061033	3067428	ERS1375019 (SAMEA4475840)
08CEB54LM	Fruit, vegetables, cereals and herbs	tomatoes	FR	2008	121	Henri et al. 2016	False	95,73	2996761	6	2996761	3058875	ERS1375020 (SAMEA4475841)
08CEB61LM	Milk and milk products	cheese	FR	2008	121	Henri et al. 2016	False	95,12	3096728	5	3096728	3097782	ERS1375022 (SAMEA4475843)
08CEB73LM	Fruit, vegetables, cereals and herbs	pineapple- fresh	FR	2008	121	Henri et al. 2016	False	95,93	2998389	9	2998389	3061149	ERS1375023 (SAMEA4475844)
09CEB328LM	Composite dishes	pate en croute	FR	2009	121	Henri et al. 2016	False	94,81	3070283	13	3070283	3136651	ERS1375030 (SAMEA4475851)
09CEB333LM	Meat and meat products	cooked chicken	FR	2009	121	Henri et al. 2016	False	95,14	3062058	1	3062058	3062058	ERS1375031 (SAMEA4475852)
09CEB351LM	Composite dishes	tomate farcies	FR	2009	121	Henri et al. 2016	False	95,25	3070738	199	3070738	3156028	ERS1375036 (SAMEA4475857)
09CEB373LM	Meat and meat products	sausage	FR	2009	121	Henri et al. 2016	False	94,72	2995793	28	2995793	3068888	ERS1375041 (SAMEA4475862)
09CEB379LM	Processing environment	surfaces	FR	2009	121	Henri et al. 2016	False	94,98	3001814	29	3001814	3074479	ERS1375042 (SAMEA4475863)
09CEB575LM	Fish and fishery products	tarama	FR	2009	121	Henri et al. 2016	False	95,15	3098158	15	3098158	3164581	ERS1375044 (SAMEA4475865)
09CEB684LM	Fish and fishery products	salmon	FR	2009	121	Henri et al. 2016	False	95,96	3064053	4	3064053	3125658	ERS1375049 (SAMEA4475870)
09CEB734LM	Meat and meat products	escalope poulet	FR	2009	121	Henri et al. 2016	False	94,98	3066434	2	3066434	3066759	ERS1375050 (SAMEA4475871)

09CEB835LM	Composite dishes	jambon persillé	FR	2009	121	Henri et al. 2016	False	95,13	3092559	11	3092559	3097109	ERS1375051 (SAMEA4475872)
09CEB864LM	Meat and meat products	lardons fumés	FR	2009	121	Henri et al. 2016	False	94,59	2958954	21	2958954	3027057	ERS1375053 (SAMEA4475874)
09CEB924LM	Meat and meat products	spliced poultry meat	FR	2009	121	Henri et al. 2016	False	95,23	3030289	45	3030289	3110220	ERS1375056 (SAMEA4475877)
10CEB339LM	Composite dishes	pork salade	FR	2010	121	Henri et al. 2016	False	96,16	3141738	36	3141738	3161160	ERS1375059 (SAMEA4475880)
10CEB630LM	Fish and fishery products	smoked salmon	FR	2010	121	Henri et al. 2016	False	96,16	3002019	22	3002019	3069021	ERS1375065 (SAMEA4475886)
10CEB631LM	Fish and fishery products	smoked salmon	FR	2010	121	Henri et al. 2016	False	95,91	3065437	8	3065437	3128299	ERS1375066 (SAMEA4475887)
11CEB367LM	Processing environment	surfaces	FR	2011	121	Henri et al. 2016	False	96	3032861	7	3032861	3095474	ERS1375085 (SAMEA4475906)
11CEB403LM	Fish and fishery products	smoked salmon	FR	2011	121	Henri et al. 2016	False	95,84	3069573	2	3069573	3094360	ERS1375088 (SAMEA4475909)
11CEB431LM	Fish and fishery products	Fish and fishery products	FR	2011	121	Fritch et al. 2019	False	95,31	3086193	3	3086193	3087055	SAMEA7290422
12CEB1306LM	Fish and fishery products	smoked trout	FR	2012	121	Henri et al. 2016	False	95,01	2995281	1	2995281	2995281	ERS1375099 (SAMEA4475920)
13CEB777LM	Fish and fishery products	smoked trout	FR	2013	121	Henri et al. 2016	False	95,98	3034253	11	3034253	3097867	ERS1375108 (SAMEA4475929)
14SEL875LM	Processing environment	#N/A	FR	2014	121	Fritch et al. 2019	False	95,24	3055756	1	3055756	3055756	ERS2647086 (SAMEA4827204)
15SEL1647LM	Composite dishes	chapelure	IE	2014	121	this study	False	95,5	3091656	6	3091656	3093399	SAMEA7290423
15SEL1648LM	Composite dishes	chapelure	IE	2014	121	this study	False	95,61	3092698	6	3092698	3095339	SAMEA7290424
15SEL1652LM	composite dishes	farce	IE	2014	121	this study	False	95,64	3090620	6	3090620	3092195	SAMEA7290425
15SEL1661LM	Composite dishes	sandwich	IE	2014	121	this study	False	95,6	3093410	2	3093410	3093810	SAMEA7290426

15SEL1665LM	Composite dishes	chapelure	IE	2014	121	this study	False	95,77	3093453	2	3093453	3093736	SAMEA7290427
15SEL1669LM	vegetable	romarin roti	IE	2012	121	this study	False	95,68	3143013	9	3143013	3147982	SAMEA7290428
15SEL1673LM	Meat and meat products	ham and chicken meat	IE	2012	121	this study	False	95,76	3148182	6	3148182	3154306	SAMEA7290429
15SEL1681LM	Composite dishes	brocoli pie	IE	2013	121	this study	False	95,8	3149222	5	3149222	3154560	SAMEA7290430
15SEL1685LM	Composite dishes	tourte viande hachée	IE	2013	121	this study	False	95,87	3098720	8	3098720	3106665	SAMEA7290431
15SEL1688LM	vegetable	vegetable	IE	2014	121	this study	False	95,91	3149062	9	3149062	3155350	SAMEA7290432
15SEL1694LM	Composite dishes	sauce	IE	2014	121	this study	False	95,6	3098419	12	3098419	3111395	SAMEA7290433
15SEL1757LM	Processing environment	lingette surface	FR	2015	121	this study	False	95,79	3090170	7	3090170	3092951	SAMEA7290434
15SEL1761LM	Processing environment	lingette	FR	2014	121	this study	False	95,78	3029016	6	3029016	3091188	SAMEA7290435
15SEL1762LM	Composite dishes	cabillaud et légume (traiteur)	FR	2011	121	this study	False	95,42	3027357	10	3027357	3095142	SAMEA7290436
15SEL701LM	Processing environment	lingette	FR	2015	121	this study	False	95,84	3001024	2	3001024	3062002	SAMEA7290437
16SEL1299LM	Wild animals	wildboar livers	DE	2012	121	this study	False	94,52	2915299	10	2915299	2981654	SAMEA7290438
16SEL787LM	Processing environment	remplisseur saucisse	MK	2012	121	this study	False	95,54	2987821	10	2987821	3054222	SAMEA7290439
16SEL791LM	Processing environment	mixieur viande	MK	2011	121	this study	False	94,48	2951143	16	2951143	3047671	SAMEA7290440
16SEL792LM	Meat and meat products	viande hachée	MK	2010	121	this study	False	94,66	2979671	8	2979671	3051840	SAMEA7290441
16SEL793LM	Meat and meat products	pork carcass	MK	2012	121	this study	False	94,26	2952047	38	2952047	3073373	SAMEA7290442
17SEL409LM	Fish and fishery products	salmon	FR	2017	121	this study	False	95,09	2544496	5	2544496	2612191	SAMEA7290443
17SEL410LM	Processing environment	lingette peleuse	FR	2017	121	this study	False	95,11	3027123	6	3027123	3095116	SAMEA7290444
17SEL47LM	Fish and fishery products	smocked salmon	FR	2017	121	this study	False	94,95	3029293	11	3029293	3093554	SAMEA7290445

17SEL48LM	Fish and fishery products	smocked salmon	FR	2017	121	this study	False	95,14	3095395	26	3095395	3101623	SAMEA7290446
17SEL64LM	Meat and meat products	merguez (beef)	FR	2017	121	this study	False	95,19	3000249	6	3000249	3068322	SAMEA7290447
18SEL586LM	Soil & farm environment	sewage water	CZ	2018	121	this study	False	94,64	2914084	5	2914084	2976083	SAMEA7290448
18SEL589LM	Soil & farm environment	sewage water	CZ	2018	121	this study	False	94,8	2964719	5	2964719	3021379	SAMEA7290449
AT-CO-M-SA-280	Fruit, vegetables, cereals and herbs	mixed food RTE - delicatessen salad, marinated, with pasta, ham and cheese	AT	2019	121	Felix et al. 2020	False	94,08	2994987	4	2994987	3056586	ERS4773663 (SAMEA7005833)
AT-CO-V-FZ-266	Fruit, vegetables, cereals and herbs	mixed food - vegan Thai-Green-Curry, frozen	AT	2016	121	Felix et al. 2020	False	94,7	3067310	26	3067310	3127038	ERS4773666 (SAMEA7005836)
AT-FI-F-FF-225	Fish and fishery products	fish - gravad salmon	AT	2016	121	Felix et al. 2020	False	94,39	3029668	6	3029668	3031715	ERS4773686 (SAMEA7005856)
AT-FI-F-FF-233	Fish and fishery products	fish - gravad salmon, sliced	AT	2018	121	Felix et al. 2020	False	92,97	2877630	33	2877630	2997611	ERS4773687 (SAMEA7005857)
AT-FI-F-SM-229	Fish and fishery products	fish - smoked salmon	AT	2017	121	Felix et al. 2020	False	93,72	3074327	15	3074327	3105139	ERS4773691 (SAMEA7005861)
AT-FI-F-SM-236	Fish and fishery products	fish - smoked salmon	AT	2018	121	Felix et al. 2020	False	93,57	2995128	19	2995128	3062373	ERS4773695 (SAMEA7005865)
AT-FI-F-SM-237	Fish and fishery products	fish - smoked salmon	AT	2018	121	Felix et al. 2020	False	94,21	3063549	18	3063549	3069890	ERS4773696 (SAMEA7005866)
AT-FI-F-SM-241	Fish and fishery products	fish - smoked salmon trout	AT	2019	121	Felix et al. 2020	False	93,44	3069267	15	3069267	3101314	ERS4773699 (SAMEA7005869)
AT-VE-M-SA-268	Fruit, vegetables, cereals and herbs	mixed food RTE - delicatessen salad	AT	2017	121	Felix et al. 2020	False	94,21	3034526	14	3034526	3101600	ERS4773725 (SAMEA7005895)

alse	Fruit, vegetables, cereals and herbs	salad RTE - salad	AT	2019	236	Felix et al. 2020	false	93,36	2984196	11	2984196	3029541	ERS4773732 (SAMEA7005902)
B7200-O2-LmUB3PA	Fish and fishery products	smoked salmon	FR	1999	121	palma et al. 2020	False	95,44	3064267	4	3064267	3125948	ERS3013737 (SAMEA5206265)
B7202-O1-LmUB3PA	Fish and fishery products	smoked salmon	FR	1999	121	palma et al. 2020	False	95,49	2961318	3	2961318	3022872	ERS3013739 (SAMEA5206267)
BE-BOV-SK-H-71	Farm animals	Hide	BE	2018	121	Felix et al. 2020	False	94,78	2937515	4	2937515	2938322	ERS4773805 (SAMEA7005975)
C154-O1-LmUB3PA	Processing environment	#N/A	FR	2000	121	palma et al. 2020	False	95,63	2959281	5	2959281	3021954	ERS3013745 (SAMEA5206273)
C5086-T1-LmUB3PA	Processing environment	#N/A	FR	2000	121	palma et al. 2020	False	95,52	2959290	4	2959290	3021424	ERS3013750 (SAMEA5206278)
C5125-O-LmUB3PA	Fish and fishery products	smoked salmon	FR	2000	121	palma et al. 2020	False	95,61	2960542	2	2960542	3021652	ERS3013751 (SAMEA5206279)
C5128-P-LmUB3PA	Fish and fishery products	smoked salmon	FR	2000	121	palma et al. 2020	False	95,5	3001392	3	3001392	3062798	ERS3013753 (SAMEA5206281)
C5134-BPL1-LmUB3PA	Fish and fishery products	smoked salmon	FR	2000	121	palma et al. 2020	False	95,53	2959442	3	2959442	3020996	ERS3013754 (SAMEA5206282)
C5205-BPL1-LmUB3PA	Fish and fishery products	smoked salmon	FR	2000	121	palma et al. 2020	False	95,58	3062929	4	3062929	3124728	ERS3013756 (SAMEA5206284)
CA2815-P-LmUB3PA	Fish and fishery products	smoked salmon	FR	2000	121	palma et al. 2020	False	95,53	2961944	10	2961944	3025981	ERS3013761 (SAMEA5206289)
CS310-S1-LmUB3PA	Processing environment	#N/A	FR	2000	121	palma et al. 2020	False	95,45	2960661	3	2960661	2961258	ERS3013782 (SAMEA5206310)
CS461-S1-LmUB3PA	Processing environment	#N/A	FR	2000	121	palma et al. 2020	False	95,47	3060581	3	3060581	3061605	ERS3013786 (SAMEA5206314)
CZ-DA-U-UN-75	Milk and milk products	dairy product	CZ	2016	121	Felix et al. 2020	False	94,13	3068050	19	3068050	3073821	ERS4773861 (SAMEA7006031)
CZ-FAR-CF-54	Soil & farm environment	strawberries from the field - field	CZ	2014	121	Felix et al. 2020	False	94,61	2929874	18	2929874	2996777	ERS4773867 (SAMEA7006037)

CZ-FI-F-UN-85	Fish and fishery products	fish product	CZ	2016	121	Felix et al. 2020	False	95,11	3097224	2	3097224	3097599	ERS4773873 (SAMEA7006043)
CZ-FI-F-UN-86	Fish and fishery products	fish product	CZ	2007	741	Felix et al. 2020	False	94,9	3039668	2	3039668	3064229	ERS4773874 (SAMEA7006044)
CZ-HU-CP-I-185	human	0	CZ	2014	121	this study	False	95,5	3059380	1	3059380	3059380	SAMEA7290450
CZ-HU-CP-I-231	human	0	CZ	2014	121	this study	False	95,52	3057159	4	3057159	3063444	SAMEA7290451
CZ-HU-CP-I-271	human	0	CZ	2016	121	this study	False	95,58	3060871	1	3060871	3060871	SAMEA7290452
CZ-ME-U-UN-119	Meat and meat products	RTE meat	CZ	2016	121	Felix et al. 2020	False	94,18	2875369	3	2875369	2936218	ERS4773892 (SAMEA7006062)
CZ-ME-U-UN-131	Meat and meat products	RTE meat	CZ	2016	121	Felix et al. 2020	False	95,04	3032488	7	3032488	3104983	ERS4773903 (SAMEA7006073)
CZ-ME-U-UN-139	Meat and meat products	RTE meat	CZ	2016	121	Felix et al. 2020	False	94,6	2949666	2	2949666	3010666	ERS4773906 (SAMEA7006076)
CZ-ME-U-UN-149	Meat and meat products	RTE meat	CZ	2019	121	Felix et al. 2020	False	94,21	2881883	3	2881883	2937666	ERS4773916 (SAMEA7006086)
CZ-ME-U-UN-153	Meat and meat products	RTE meat	CZ	2014	121	Felix et al. 2020	False	95,14	3003676	4	3003676	3065580	ERS4773920 (SAMEA7006090)
CZ-VE-F-102	Fruit, vegetables, cereals and herbs	fruit - fresh	CZ	2014	121	Felix et al. 2020	False	94,43	2932806	3	2932806	2994003	ERS4773961 (SAMEA7006131)
DE-WBO-UN-34	Wild animals	wild boar	DE	2012	121	Felix et al. 2020	False	94,52	2916667	9	2916667	2982509	ERS4774021 (SAMEA7006192)
DN-CO-M-50	Composite dishes	Composite dish - Tzatziki (greek style, cucumber and yoghurt)	DN	2018	121	Felix et al. 2020	False	94,27	3058579	36	3058579	3075970	ERS4774023 (SAMEA7006194)
DN-CO-M-54	Composite dishes	Composite dish - Mustard mayonnaise	DN	2014	236	Felix et al. 2020	False	95,38	3007951	2	3007951	3069147	ERS4774025 (SAMEA7006196)
DN-CO-M-67	Composite dishes	Composite dish - Chicken salad	DN	2015	121	Felix et al. 2020	False	95,3	3066642	22	3066642	3137400	ERS4774029 (SAMEA7006200)
DN-CO-M-77	Composite dishes	Composite dish - Sandwich with salmon/green salad	DN	2016	121	Felix et al. 2020	False	95,61	3095118	32	3095118	3108757	ERS4774034 (SAMEA7006205)
DN-FI-F-FF-70	Fish and fishery products	RTE fish - Salmon	DN	2015	121	Felix et al. 2020	False	95,33	3035574	3	3035574	3097054	ERS4774037 (SAMEA7006208)

DN-FI-F-SM-43	Fish and fishery products	RTE fish - Smoked salmon	DN	2017	121	Felix et al. 2020	False	94,29	2999991	53	2999991	3083318	ERS4774041 (SAMEA7006212)
DN-FI-F-SM-46	Fish and fishery products	RTE fish - Smoked salmon	DN	2017	121	Felix et al. 2020	False	95,09	3098680	2	3098680	3098931	ERS4774044 (SAMEA7006215)
DN-FI-F-SM-58	Fish and fishery products	RTE fish - Smoked halibut	DN	2014	121	Felix et al. 2020	False	95,43	3099193	2	3099193	3099442	ERS4774046 (SAMEA7006217)
DN-FI-F-SM-71	Fish and fishery products	RTE fish - Smoked salmon	DN	2015	121	Felix et al. 2020	False	95,41	3226793	9	3226793	3229685	ERS4774049 (SAMEA7006220)
DN-ME-B-PM-78	Meat and meat products	RTE meat - Roasted beef, sliced	DN	2017	121	Felix et al. 2020	False	95,04	3101051	29	3101051	3113590	ERS4774056 (SAMEA7006227)
DN-ME-P-DE-32	Meat and meat products	RTE meat - Pork salami	DN	2015	236	Felix et al. 2020	False	95,26	3027733	2	3027733	3028185	ERS4774061 (SAMEA7006232)
DN-ME-P-PM-10	Meat and meat products	RTE meat - Rolled seasoned meat (pork), sliced	DN	2017	121	Felix et al. 2020	False	94,98	3097489	33	3097489	3111172	ERS4774066 (SAMEA7006237)
DN-ME-P-PM-8	Meat and meat products	RTE meat - Rolled seasoned meat (pork), sliced	DN	2017	121	Felix et al. 2020	False	94,45	3097910	94	3097910	3138545	ERS4774073 (SAMEA7006244)
DN-ME-P-U-31	Meat and meat products	RTE meat - Chicken	DN	2015	121	Felix et al. 2020	False	95,31	3038356	11	3038356	3102179	ERS4774075 (SAMEA7006246)
DN-ME-UN-DE-25	Meat and meat products	RTE meat - Meatballs	DN	2015	121	Felix et al. 2020	False	95,54	3037281	8	3037281	3101950	ERS4774076 (SAMEA7006247)
FI-PIG-CP-U-141	Farm animals	Pig - Tonsil	FI	2003	121	Felix et al. 2020	False	94,94	2880714	3	2880714	2881382	ERS4774280 (SAMEA7006451)
FR-CO-M-OT-599	Composite dishes	Plats cuisinés - nem chua BLA	FR	2017	121	Felix et al. 2020	False	94,9	3025808	3	3025808	3032190	ERS4774306 (SAMEA7006477)
FR-CO-M-OT-600	Composite dishes	Plats cuisinés - farce pour pâtes crues	FR	2017	121	Felix et al. 2020	False	94,07	2934175	179	2934175	3048364	ERS4774307 (SAMEA7006478)
FR-CO-M-OT-604	Composite dishes	Plats cuisinés - nem chua BLA	FR	2017	121	Felix et al. 2020	False	94,82	2963529	214	2963529	3091478	#N/A
FR-CO-M-SA-708	Composite dishes	Plats cuisinés - saladecomposéepouletoeufdurtomatesalade	FR	2011	121	Henri et al. 2016	False	96,03	2997886	5	2997886	3059898	ERS4774319 (SAMEA7006490)
FR-DA-U-CH-638	Milk and milk products	Lait et fromage - fromage non affiné au lait pasteurisé	FR	2017	121	Felix et al. 2020	False	94,23	2932719	3	2932719	2994273	ERS4774346 (SAMEA7006517)
FR-FI-F-FF-615	Fish and fishery products	Poisson - dos de cabillaud	FR	2017	121	Felix et al. 2020	False	95,2	3033852	3	3033852	3095542	ERS4774396 (SAMEA7006567)

FR-FI-F-SM-611	Fish and fishery products	Poisson - filets saumon fumé à cuire	FR	2017	121	Felix et al. 2020	False	95,16	3094292	14	3094292	3097567	ERS4774422 (SAMEA7006593)
FR-FI-F-SM-621	Fish and fishery products	Poisson - saumon fumé	FR	2017	121	Felix et al. 2020	False	95,09	3027759	5	3027759	3095508	ERS4774423 (SAMEA7006594)
FR-FI-F-SM-627	Fish and fishery products	Poisson - saumon fumé	FR	2017	121	Felix et al. 2020	False	94,97	3150408	34	3150408	3228661	ERS4774424 (SAMEA7006595)
FR-FI-F-SM-642	Fish and fishery products	Poisson - saumon fumé ecosse	FR	2017	121	Felix et al. 2020	False	95,22	3026808	7	3026808	3095430	ERS4774425 (SAMEA7006596)
FR-FI-S-OT-629	Fish and fishery products	Crustacés - cocktail fruits de mer	FR	2017	121	Felix et al. 2020	False	95,09	3105919	9	3105919	3111073	ERS4774434 (SAMEA7006605)
FR-FI-U-UN-440	Fish and fishery products	Fish and fishery products	FR	2008	121	Fritch et al. 2019	False	95,4	3044157	5	3044157	3111502	ERR2727616 (SAMEA4816501)
FR-FI-U-UN-447	Fish and fishery products	Fish and fishery products	FR	2010	121	Fritch et al. 2019	False	95,11	2982985	3	2982985	2983553	ERR2727620 (SAMEA4816505)
FR-FI-U-UN-450	Fish and fishery products	Fish and fishery products	FR	2010	121	Fritch et al. 2019	False	95,43	2993100	4	2993100	3060115	ERR2727621 (SAMEA4816507)
FR-FI-U-UN-462	Fish and fishery products	Fish and fishery products	FR	2011	121	Fritch et al. 2019	False	95,45	3111620	9	3111620	3117270	ERR2728039 (SAMEA4819877)
FR-FI-U-UN-476	Fish and fishery products	Fish and fishery products	FR	2011	121	Typelipo	False	95,73	3090381	10	3090381	3094922	ERS4774437 (SAMEA7006608)
FR-ME-A-DE-617	Meat and meat products	Charcuterie - foie gras	FR	2017	121	Felix et al. 2020	False	94,55	2952366	5	2952366	3020082	ERS4774443 (SAMEA7006614)
FR-ME-A-DE-672	Meat and meat products	Volaille - foie de volaille	FR	2017	121	Felix et al. 2020	False	95,01	3059959	6	3059959	3063831	ERS4774447 (SAMEA7006618)
FR-ME-B-DE-648	Meat and meat products	Charcuterie - merguez de bœuf	FR	2017	121	Felix et al. 2020	False	94,9	3005254	4	3005254	3067663	ERS4774453 (SAMEA7006624)
FR-ME-B-PM-614	Meat and meat products	Bovin - steack haché	FR	2017	121	Felix et al. 2020	False	94,54	3013754	4	3013754	3076071	ERS4774456 (SAMEA7006627)

FR-ME-P-PM-658	Meat and meat products	Charcuterie - échine de porc	FR	2017	121	Felix et al. 2020	False	95,05	3026217	5	3026217	3093326	ERS4774488 (SAMEA7006659)
FR-ME-U-UN-448	Meat and meat products	Meat and meat products	FR	2010	121	Henri et al. 2016	False	94,87	3020961	31	3020961	3094999	ERR1738661 (SAMEA4475883)
FR-ME-U-UN-452	Meat and meat products	Meat and meat products	FR	2011	121	Henri et al. 2016	False	95,5	2960977	16	2960977	3025981	ERR1738769 (SAMEA4475892)
FR-ME-U-UN-454	Meat and meat products	Meat and meat products	FR	2011	121	Henri et al. 2016	False	96,11	2999948	8	2999948	3062681	ERR1738776 (SAMEA4475899)
FR-ME-U-UN-467	Meat and meat products	Meat and meat products	FR	2012	121	Henri et al. 2016	False	95,36	2993813	3	2993813	3055047	ERR1738800 (SAMEA4475923)
FR-PE-FI-M-654	Composite dishes	Poisson - lingette: cuve tarama	FR	2017	121	Felix et al. 2020	False	94,93	3024428	2	3024428	3025008	ERS4774522 (SAMEA7006693)
FR-PE-ME-S-620	Meat and meat products	Volaille - surface	FR	2017	121	Felix et al. 2020	False	95,28	3033673	2	3033673	3094783	ERS4774523 (SAMEA7006694)
FR-VE-F-OT-594	Fruit, vegetables, cereals and herbs	Plats cuisinés - purée de céleri	FR	2017	121	Felix et al. 2020	False	94,9	3083076	4	3083076	3089547	ERS4774528 (SAMEA7006699)
FR-VE-U-UN-436	Fruit, vegetables, cereals and herbs	Fruit, vegetables, cereals and herbs	FR	2008	121	Henri et al. 2016	False	96,03	3670322	15	3670322	3674491	ERS4774533 (SAMEA7006704)
FR-VE-U-UN-469	Fruit, vegetables, cereals and herbs	Fruit, vegetables, cereals and herbs	FR	2012	121	Henri et al. 2016	False	95,83	3122284	7	3122284	3184895	ERR1738796 (SAMEA4475919)
FR-VE-V-FZ-711	Fruit, vegetables, cereals and herbs	fruits rougessurgelé - fruits rouges surgelés	FR	2011	121	Henri et al. 2016	False	96,24	3004765	8	3004765	3006535	ERS4774540 (SAMEA7006711)
FR-VE-V-OT-601	Fruit, vegetables, cereals and herbs	Plats cuisinés - crème de brocolis	FR	2017	121	Felix et al. 2020	False	94,9	3092697	81	3092697	3115081	ERS4774545 (SAMEA7006716)
FR-VE-V-RA-700	Fruit, vegetables, cereals and herbs	légumes - surface	FR	2012	121	Henri et al. 2016	False	95,42	3012491	12	3012491	3077963	ERS4774557 (SAMEA7006728)

FR-VE-V-RA-701	Fruit, vegetables, cereals and herbs	légumes - surface	FR	2012	121	Henri et al. 2016	False	95,36	2990476	9	2990476	3053560	ERS4774558 (SAMEA7006729)
FR-VE-V-RA-709	Fruit, vegetables, cereals and herbs	Plats cuisinés - carottesrâpéessaladevinaigrette	FR	2011	121	Henri et al. 2016	False	95,49	2936682	27	2936682	3004385	ERS4774560 (SAMEA7006731)
FR-VE-V-RA-725	Fruit, vegetables, cereals and herbs	légumes - Tomates crues	FR	2006	121	Henri et al. 2016	False	95,77	3121687	5	3121687	3122718	ERS4774564 (SAMEA7006735)
FR-VE-V-SA-723	Fruit, vegetables, cereals and herbs	légumes - poireaux salade	FR	2008	121	Henri et al. 2016	False	96,08	3061892	16	3061892	3126674	ERS4774567 (SAMEA7006738)
FR-VE-V-SA-724	Fruit, vegetables, cereals and herbs	légumes - endives en salade	FR	2008	121	Henri et al. 2016	False	95,85	2998290	3	2998290	3059891	ERS4774568 (SAMEA7006739)
FSL F3-0293	Soil & farm environment	mangeoir	US	2003	121	Fugett et al. 2007	False	95,26	2916016	3	2916016	2916496	SRR12518428
FSL F3-146	Soil & farm environment	water (farm)	US	2003	121	Fugett et al. 2007	False	94,5	2878778	3	2878778	2884439	SRR12164826
FSL F3-194	Farm animals	bovine (feces)	US	2003	121	Fugett et al. 2007	False	94,57	2878774	5	2878774	2884790	SRR12518435
HR-BOV-CP-I-46	Farm animals	Abortion Cattle	HR	2016	121	Felix et al. 2020	False	95,57	3007019	4	3007019	3068179	ERS4774577 (SAMEA7006748)
HR-BOV-CP-I-47	Farm animals	Abortion Cattle	HR	2016	121	Felix et al. 2020	False	95,57	3067108	2	3067108	3067599	ERS4774578 (SAMEA7006749)
IT-CO-U-UN-146	Composite dishes	composite dishes - - composite dishes	IT	2016	121	Felix et al. 2020	false	95,01	3077851	32	3077851	3146450	ERS4774598 (SAMEA7006769)
IT-FI-F-UN-223	Fish and fishery products	SALMON - - Fish and fishery products	IT	2015	121	Felix et al. 2020	False	94,49	3053432	1	3053432	3053432	ERS4774645 (SAMEA7006816)
IT-FI-F-UN-234	Fish and fishery products	SALMON - - Fish and fishery products	IT	2014	121	Felix et al. 2020	False	94,79	3123484	5	3123484	3126846	ERS4774653 (SAMEA7006824)

IT-FI-F-UN-238	Fish and fishery products	TUNA - - Fish and fishery products	IT	2015	121	Felix et al. 2020	False	94,92	3061732	2	3061732	3122829	ERS4774657 (SAMEA7006828)
IT-FI-F-UN-240	Fish and fishery products	SALMON - - Fish and fishery products	IT	2014	121	Felix et al. 2020	False	95,18	3032414	60	3032414	3107956	ERS4774658 (SAMEA7006829)
IT-FI-F-UN-241	Fish and fishery products	SALMON - - Fish and fishery products	IT	2014	121	Felix et al. 2020	False	94,82	3070193	30	3070193	3102089	ERS4774659 (SAMEA7006830)
IT-FI-F-UN-251	Fish and fishery products	SALMON (Salmo salar) - - Fish and fishery products	IT	2019	121	Felix et al. 2020	False	95,5	3029489	3	3029489	3090817	ERS4774666 (SAMEA7006837)
IT-FI-S-UN-239	Fish and fishery products	MOLLUSCS - - Fish and fishery products	IT	2014	121	Felix et al. 2020	False	94,92	3061732	2	3061732	3122829	ERS4774669 (SAMEA7006840)
IT-ME-B-RA-279	Meat and meat products	Raw meat - Bovine - Meat and meat products	IT	2014	121	Felix et al. 2020	False	95,12	3065303	2	3065303	3065595	ERS4774688 (SAMEA7006859)
IT-ME-P-DE-265	Meat and meat products	SOPPRESSATA - Pork - Meat and meat products	IT	2016	121	Felix et al. 2020	False	95,22	3057361	4	3057361	3058547	ERS4774699 (SAMEA7006870)
IT-ME-P-DE-271	Meat and meat products	MORTADELLA - Pork - Meat and meat products	IT	2015	121	Felix et al. 2020	False	94,71	2969961	2	2969961	2970170	ERS4774704 (SAMEA7006875)
IT-ME-P-DE-288	Meat and meat products	salami - Pork - Meat and meat products	IT	2016	121	Felix et al. 2020	False	94,6	3062662	27	3062662	3069059	ERS4774713 (SAMEA7006884)
IT-ME-P-DE-290	Meat and meat products	Sausage - Pork - Meat and meat products	IT	2016	121	Felix et al. 2020	False	94,69	3034449	3	3034449	3095786	ERS4774715 (SAMEA7006886)
IT-NAT-SO-53	Soil & farm environment	- Environment	IT	2018	121	Felix et al. 2020	False	95,04	3030428	31	3030428	3099846	ERS4774730 (SAMEA7006901)
IT-OTH-CP-36	Wild animals	Dolphin - Brain	IT	2017	121	Felix et al. 2020	False	95,26	2967551	11	2967551	3030518	ERS4775211 (SAMEA7007383)
IT-PE-ME-U-286	Processing environment	Swab (SPONGE BAGS) - - Meat and meat products	IT	2016	121	Felix et al. 2020	False	95,53	3096852	5	3096852	3097986	ERS4774777 (SAMEA7006949)
K7970-LmUB3PA	Fish and fishery products	shrimp	FR	2008	121	palma et al. 2020	False	94,71	2933729	4	2933729	2995314	ERS3013810 (SAMEA5206338)
K8125-LmUB3PA	Processing environment	#N/A	FR	2008	121	palma et al. 2020	False	94,74	2994343	3	2994343	2995018	ERS3013811 (SAMEA5206339)
K8510-LmUB3PA	Fish and fishery products	shrimp	FR	2008	121	palma et al. 2020	False	94,71	2994115	2	2994115	2994559	ERS3013812 (SAMEA5206340)

K8642-LmUB3PA	Processing environment	#N/A	FR	2008	121	palma et al. 2020	False	94,7	2994253	2	2994253	2994697	ERS3013813 (SAMEA5206341)
L1017-LmUB3PA	Fish and fishery products	shrimp	FR	2009	121	palma et al. 2020	False	94,75	2993897	2	2993897	2994341	ERS3013814 (SAMEA5206342)
L7561-LmUB3PA	Processing environment	#N/A	FR	2009	121	palma et al. 2020	False	94,74	3036044	4	3036044	3097998	ERS3013815 (SAMEA5206343)
L-893-LmUB3PA	Processing environment	#N/A	FR	2009	121	palma et al. 2020	False	94,73	2932621	3	2932621	2933310	ERS3013816 (SAMEA5206344)
L9562-LmUB3PA	Fish and fishery products	shrimp	FR	2009	121	palma et al. 2020	False	94,71	2994018	3	2994018	2994707	ERS3013817 (SAMEA5206345)
M129-LmUB3PA	Fish and fishery products	Shrimp	FR	2010	121	palma et al. 2020	False	94,75	2993655	4	2993655	2994437	ERS3013818 (SAMEA5206346)
M5372-LmUB3PA	Fish and fishery products	Shrimp	FR	2010	121	palma et al. 2020	False	94,72	2932731	4	2932731	2994413	ERS3013819 (SAMEA5206347)
M5596-LmUB3PA	Fish and fishery products	Environment	FR	2010	121	palma et al. 2020	False	94,64	2995261	4	2995261	2996512	ERS3013820 (SAMEA5206348)
M896-LmUB3PA	Fish and fishery products	Environment	FR	2010	121	palma et al. 2020	False	94,75	2933361	3	2933361	2994716	ERS3013821 (SAMEA5206349)
N44-LmUB3PA	Fish and fishery products	Shrimp	FR	2011	121	palma et al. 2020	False	94,72	2934840	4	2934840	2996504	ERS3013822 (SAMEA5206350)
N7764-LmUB3PA	Fish and fishery products	Environment	FR	2011	121	palma et al. 2020	False	94,71	2994020	2	2994020	2994464	ERS3013823 (SAMEA5206351)
N8001-LmUB3PA	Fish and fishery products	Shrimp	FR	2011	121	palma et al. 2020	False	94,7	2995102	2	2995102	2995386	ERS3013824 (SAMEA5206352)
N8038-LmUB3PA	Fish and fishery products	Environment	FR	2011	121	palma et al. 2020	False	94,72	2994417	6	2994417	2996281	ERS3013825 (SAMEA5206353)
NL-AVI-UN-36	Farm animals	chicken	NL	2017	121	Felix et al. 2020	False	95,77	3130729	1	3130729	3130729	ERS4774816 (SAMEA7006988)

NL-AVI-UN-37	Farm animals	chicken	NL	2018	121	Felix et al. 2020	False	95,77	3052579	4	3052579	3114485	ERS4774817 (SAMEA7006989)
O116-LmUB3PA	Fish and fishery products	Environment	FR	2012	121	palma et al. 2020	False	94,7	2994236	4	2994236	2995524	ERS3013826 (SAMEA5206354)
O228-LmUB3PA	Fish and fishery products	Shrimp	FR	2012	121	palma et al. 2020	False	94,75	2994130	4	2994130	2995248	ERS3013827 (SAMEA5206355)
PL-URB-PA-5	Soil & farm environment	Caspian town Park - soil	PL	2015	593	Felix et al. 2020	False	95,23	2980157	18	2980157	2985396	ERS4774935 (SAMEA7007107)
RL15000006	Fish and fishery products	Fish Smoked (Salmon)	FR	2010	121	Painset et al. 2019	False	95,74	2959786	6	2959786	3021593	SRR7440649
RL15000009	Fish and fishery products	Fish Smoked (Salmon)	FR	2011	121	Painset et al. 2019	False	95,88	3004106	4	3004106	3066238	SRR7440652
RL15000010	Meat and meat products	Mixed sources Deli products - Pate Cooked	FR	2011	121	Painset et al. 2019	False	94,62	3040729	16	3040729	3077703	SRR7440645
RL15000011	Fish and fishery products	Fish (Salmon)	FR	2011	121	Painset et al. 2019	False	94,18	3035461	47	3035461	3110544	SRR7440646
RL15000014	Fish and fishery products	Fish Smoked (Salmon)	FR	2011	121	Painset et al. 2019	False	95,84	3032955	5	3032955	3096379	SRR7440654
RL15000015	Fish and fishery products	Fish Wild fish Smoked (Herring, kipper)	FR	2011	121	Painset et al. 2019	False	94,81	3047513	14	3047513	3082196	SRR7440655
RL15000018	Fish and fishery products	Fish Wild fish Filet (Herring, kipper)	FR	2011	121	Painset et al. 2019	False	95,82	2993092	7	2993092	3055567	SRR7441209
RL15000019	Fish and fishery products	Fish Smoked (Salmon)	FR	2011	121	Painset et al. 2019	False	94,53	3006710	32	3006710	3078631	SRR7441208
RL15000023	Fish and fishery products	Fish Smoked (Salmon)	FR	2011	121	Painset et al. 2019	False	96,04	3031967	5	3031967	3095550	SRR7441212
RL15000026	Meat and meat products	Unspecified Deli products - Pate	LV	2011	121	Painset et al. 2019	False	94,91	2930863	3	2930863	2991884	SRR7440949

RL15000031	Fish and fishery products	Fish Cold smoked	BG	2011	121	Painset et al. 2019	false	95,18	3018920	5	3018920	3021619	SRR7441023
RL15000038	Fish and fishery products	Fish Gravad/slightly salted	BG	2011	121	Painset et al. 2019	False	95,08	2955245	9	2955245	3021360	SRR7440588
RL15000042	Fish and fishery products	Fish Cold smoked	BG	2011	121	Painset et al. 2019	false	93,86	2936279	15	2936279	2966530	SRR7440584
RL15000051	Fish and fishery products	Fish Wild fish Cold smoked	HU	2011	121	Painset et al. 2019	False	94,64	3035207	3	3035207	3060080	SRR7440829
RL15000055	Fish and fishery products	Fish Wild fish Warm smoked	HU	2011	121	Painset et al. 2019	False	93,49	3021047	18	3021047	3054649	SRR7440817
RL15000060	Fish and fishery products	Fish Wild fish Gravad/slightly salted	LT	2011	121	Painset et al. 2019	true	95,85	3031933	10	3031933	3096792	SRR7440906
RL15000061	Fish and fishery products	Fish Smoked	UK	2010	121	Painset et al. 2019	false	95,26	3025936	8	3025936	3095096	SRR7440905
RL15000062	Fish and fishery products	Fish Smoked	UK	2010	121	Painset et al. 2019	False	95,19	3033296	5	3033296	3095348	SRR7440904
RL15000063	Fish and fishery products	Fish Gravad/slightly salted	UK	2011	121	Painset et al. 2019	False	95,42	3002088	6	3002088	3063711	SRR7440903
RL15000085	Fish and fishery products	Fish Smoked	PL	2011	121	Painset et al. 2019	False	95,79	2957298	5	2957298	3019090	SRR7440684
RL15000091	Fish and fishery products	Fish Cold smoked	PL	2011	121	Painset et al. 2019	False	94,63	2914582	10	2914582	2985512	SRR7441147
RL15000093	Fish and fishery products	Fish Cold smoked	PL	2011	121	Painset et al. 2019	False	95,03	3016536	15	3016536	3052664	SRR7441149
RL15000098	Fish and fishery products	Fish Warm smoked	PL	2011	121	Painset et al. 2019	False	94,73	3040154	26	3040154	3083510	SRR7440709

RL15000121	Fish and fishery products	Fish Cold smoked	PL	2011	121	Painset et al. 2019	False	95,14	2995042	7	2995042	2998921	SRR7440703
RL15000128	Fish and fishery products	Fish	BE	2011	121	Painset et al. 2019	False	95,87	3032300	6	3032300	3096151	SRR7440558
RL15000129	Fish and fishery products	Fish	BE	2011	121	Painset et al. 2019	False	95,37	3031812	8	3031812	3095592	SRR7440561
RL15000130	Fish and fishery products	Fish	BE	2011	121	Painset et al. 2019	false	95,82	3000561	168	3000561	3111839	SRR7440560
RL15000134	Fish and fishery products	Fish Cold smoked	NL	2011	121	Painset et al. 2019	False	95,88	3002061	8	3002061	3068143	SRR7440564
RL15000142	Fish and Fishery products	Fish Cold smoked	NL	2011	121	Painset et al. 2019	False	94,46	2913374	18	2913374	2947563	SRR7441009
RL15000145	Fish and fishery products	Fish Smoked	NL	2011	121	Painset et al. 2019	true	94,8	2995919	32	2995919	3066336	SRR7440996
RL15000148	Fish and fishery products	Fish Wild fish Gravad/slightly salted	NL	2011	121	Painset et al. 2019	False	95,59	3031703	4	3031703	3093005	SRR7440843
RL15000154	Fish and fishery products	Fish Wild fish Smoked	DN	2010	121	Painset et al. 2019	False	94,72	3046946	19	3046946	3078057	SRR7440845
RL15000156	Fish and fishery products	Fish Wild fish Smoked	DN	2011	121	Painset et al. 2019	False	93,89	2998780	19	2998780	3088165	SRR7440835
RL15000158	Fish and fishery products	Fish Cold smoked	IE	2011	121	Painset et al. 2019	false	94,34	3032850	19	3032850	3068613	SRR7441204
RL15000160	Fish and fishery products	Fish Smoked	IE	2011	121	Painset et al. 2019	False	95,83	3033680	6	3033680	3099044	SRR7440605
RL15000161	Meat and meat products	Gallus gallus (fowl) Fresh meat - Cut Raw	FR	2011	121	Painset et al. 2019	False	95,6	3002983	7	3002983	3068316	SRR7440598

RL15000163	Fish and fishery products	Fish Raw (Salmon)	FR	2011	121	Painset et al. 2019	False	95,73	3019440	11	3019440	3085984	SRR7440600
RL15000165	Fish and fishery products	Fish (Salmon)	FR	2011	121	Painset et al. 2019	false	95,08	2995119	5	2995119	3060178	SRR7440594
RL15000166	Fish and fishery products	Fish Raw (Salmon)	FR	2011	121	Painset et al. 2019	False	95,88	3033867	6	3033867	3098320	SRR7441200
RL15000167	Fish and fishery products	Fish Raw (Trout)	FR	2011	121	Painset et al. 2019	False	95,47	3032852	5	3032852	3095897	SRR7441144
RL15000168	Fish and fishery products	Fish Raw (Salmon)	FR	2011	121	Painset et al. 2019	False	95,31	3018136	15	3018136	3089666	SRR7441143
RL15000169	Fish and fishery products	Fish Raw (Salmon)	FR	2011	121	Painset et al. 2019	False	95	2958669	11	2958669	3020567	SRR7441146
RL15000170	Fish and fishery products	Fish Raw (Salmon)	FR	2011	121	Painset et al. 2019	False	94,3	2963597	34	2963597	3034528	SRR7441145
RL15000173	Fish and fishery products	Fish Cold smoked	EE	2011	121	Painset et al. 2019	False	95,94	3033451	5	3033451	3095473	SRR7441142
RL15000174	Fish and fishery products	Fish Wild fish Warm smoked	EE	2011	121	Painset et al. 2019	false	94,44	2950127	9	2950127	3020100	SRR7441141
RL15000178	Meat and meat products	Fish Deli product - Sliced Cooked (Salmon)	EL	2011	121	Painset et al. 2019	False	95,26	3065226	6	3065226	3096302	SRR7440937
RL15000181	Fish and fishery products	Fish Warm smoked	SK	2011	121	Painset et al. 2019	false	94,73	2947786	205	2947786	3071789	SRR7440932
RL15000186	Fish and fishery products	Fish Farmed fish Gravad/slightly salted	AT	2011	121	Painset et al. 2019	False	94,67	2974212	70	2974212	3105227	SRR7440940
RL15000188	Fish and fishery products	Fish Farmed fish Smoked	AT	2011	121	Painset et al. 2019	False	94,23	2970710	16	2970710	3079273	SRR7441018

RL15000190	Fish and fishery products	Fish Cold smoked	SI	2011	121	Painset et al. 2019	False	95,9	3097603	5	3097603	3098627	SRR7441016
RL15000197	Fish and fishery products	Fish Cold smoked	DE	2010	121	Painset et al. 2019	false	95,83	3061561	5	3061561	3064342	SRR7441283
RL15000201	Fish and fishery products	Fish Smoked	DE	2010	121	Painset et al. 2019	false	95,84	3034238	9	3034238	3098752	SRR7441287
RL15000202	Meat and meat products	Swine Deli products - Pate Cooked	DE	2010	236	Painset et al. 2019	False	95,89	3001080	5	3001080	3064517	SRR7441288
RL15000203	Meat and meat products	Swine Deli products - Pate Cooked	DE	2010	236	Painset et al. 2019	False	95,8	2993814	6	2993814	3057733	SRR7441289
RL15000206	Fish and fishery products	Fish Cold smoked	DE	2010	121	Painset et al. 2019	False	95,86	3028439	7	3028439	3095434	SRR7441276
RL15000208	Fish and fishery products	Fish Cold smoked	DE	2010	121	Painset et al. 2019	False	95,82	3061008	6	3061008	3065357	SRR7441037
RL15000214	Fish and fishery products	Fish Cold smoked	DE	2010	121	Painset et al. 2019	False	95,88	3028541	7	3028541	3095570	SRR7441031
RL15000217	Fish and fishery products	Fish Warm smoked	DE	2010	121	Painset et al. 2019	False	95,94	3036739	5	3036739	3099537	SRR7440595
RL15000218	Fish and fishery products	Fish Cold smoked	DE	2010	121	Painset et al. 2019	False	95,87	2999939	5	2999939	3062194	SRR7440863
RL15000219	Fish and fishery products	Fish Smoked	DE	2010	121	Painset et al. 2019	False	95,85	3000581	5	3000581	3062657	SRR7440597
RL15000223	Fish and fishery products	Fish Cold smoked	DE	2010	121	Painset et al. 2019	False	95,83	2994889	10	2994889	3057976	SRR7440593
RL15000224	Fish and fishery products	Fish Warm smoked	DE	2010	121	Painset et al. 2019	False	95,89	3033323	6	3033323	3097136	SRR7440592

RL15000225	Fish and fishery products	Fish Warm smoked	DE	2010	121	Painset et al. 2019	False	95,95	3035250	4	3035250	3096533	SRR7440603
RL15000226	Fish and fishery products	Fish Cold smoked	DE	2010	121	Painset et al. 2019	False	95,97	3026962	7	3026962	3090649	SRR7441203
RL15000229	Fish and fishery products	Fish Cold smoked	DE	2010	121	Painset et al. 2019	False	95,88	2999830	7	2999830	3062533	SRR7440753
RL15000230	Fish and fishery products	Fish Cold smoked	DE	2010	121	Painset et al. 2019	False	95,92	3034269	8	3034269	3096975	SRR7440754
RL15000231	Fish and fishery products	Fish Warm smoked	DE	2010	121	Painset et al. 2019	False	95,91	3033155	6	3033155	3095705	SRR7440747
RL15000233	Fish and fishery products	Fish Cold smoked	DE	2010	121	Painset et al. 2019	False	95,77	3018090	10	3018090	3089260	SRR7440749
RL15000236	Fish and fishery products	Fish Smoked	DE	2011	121	Painset et al. 2019	False	95,88	2996098	10	2996098	3064552	SRR7440745
RL15000237	Fish and fishery products	Fish Cold smoked	DE	2011	121	Painset et al. 2019	False	94,34	2892058	18	2892058	2957521	SRR7441301
RL15000239	Fish and fishery products	Fish Smoked	DE	2011	121	Painset et al. 2019	False	95,93	3031147	7	3031147	3094984	SRR7441299
RL15000240	Fish and fishery products	Fish Cold smoked	DE	2011	121	Painset et al. 2019	false	95,95	3028390	4	3028390	3090006	SRR7441298
RL15000241	Fish and fishery products	Fish Warm smoked	DE	2011	121	Painset et al. 2019	False	95,93	3031871	3	3031871	3093156	SRR7441305
RL15000242	Meat and meat products	Swine Deli product - Sausage Cooked	DE	2011	236	Painset et al. 2019	False	95,77	2989139	9	2989139	3053490	SRR7441304
RL15000243	Meat and meat products	Swine Deli product - Sausage Cooked	DE	2011	121	Painset et al. 2019	False	95,8	2987988	4	2987988	3049128	SRR7441303

RL15000244	Fish and fishery products	Fish Cold smoked	DE	2011	121	Painset et al. 2019	False	94,95	2932615	4	2932615	2995798	SRR7441302
RL15000246	Fish and fishery products	Fish Farmed fish Gravad/slightly salted	SE	2010	121	Painset et al. 2019	False	95,17	2912660	4	2912660	2974246	SRR7441296
RL15000251	Fish and fishery products	Fish Farmed fish Gravad/slightly salted	SE	2010	121	Painset et al. 2019	False	96,06	3029698	7	3029698	3092489	SRR7441137
RL15000252	Fish and fishery products	Fish Smoked (process not specify)	IT	2011	121	Painset et al. 2019	False	95,93	3028310	8	3028310	3093481	SRR7441138
RL15000253	Fish and fishery products	Fish Farmed fish Smoked (process not specify)	IT	2011	121	Painset et al. 2019	False	95,88	3031526	8	3031526	3095394	SRR7441135
RL15000262	Fish and fishery products	Fish Farmed fish Smoked (process not specify)	IT	2011	121	Painset et al. 2019	False	95,88	2962388	8	2962388	3028604	SRR7440695
RL15000269	Fish and fishery products	Fish Farmed fish Gravad/slightly salted	SE	2010	121	Painset et al. 2019	False	95,84	3003247	4	3003247	3064845	SRR7440606
RL15000276	Fish and fishery products	Fish Farmed fish Smoked (process not specify)	IT	2011	121	Painset et al. 2019	False	95,84	2999014	7	2999014	3064234	SRR7440618
RL15000277	Fish and fishery products	Fish Farmed fish Gravad/slightly salted	SE	2010	121	Painset et al. 2019	False	95,08	2912547	6	2912547	2974695	SRR7440929
RL15000282	Fish and fishery products	Fish Farmed fish Warm smoked	IT	2011	121	Painset et al. 2019	False	95,81	3033211	5	3033211	3096694	SRR7440924
RL15000284	Fish and fishery products	Fish	IT	2011	121	Painset et al. 2019	False	95,89	3073920	4	3073920	3135536	SRR7440923
RL15000287	Fish and fishery products	Fish and fishery products Cold smoked	NO	2010	121	Painset et al. 2019	False	95,88	2994006	6	2994006	3056017	SRR7440921
RL15000289	Fish and fishery products	Fish and fishery products Cold smoked	NO	2010	121	Painset et al. 2019	False	95,84	2991015	9	2991015	3056126	SRR7440615

RL15000296	Fish and fishery products	Fish Warm smoked	FI	2010	121	Painset et al. 2019	False	95,73	3002554	7	3002554	3066398	SRR7440610
RL15000301	Fish and fishery products	Fish Gravad/slightly salted	FI	2010	121	Painset et al. 2019	False	95,1	2953657	7	2953657	3018368	SRR7440860
RL15000333	Fish and fishery products	Fish Warm smoked	DE	2011	121	Painset et al. 2019	False	95,82	2997788	9	2997788	3063939	SRR7440911
RL15000336	Fish and fishery products	Fish	FI	2010	121	Painset et al. 2019	False	95,2	2954571	6	2954571	3018837	SRR7440916
RL15000347	Meat and meat products	Swine Deli product - Ham	CY	2011	121	Painset et al. 2019	False	95,89	2996377	4	2996377	3058507	SRR7440548
RL15000360	Meat and meat products	Gallus gallus (fowl) Meat - Cut	NL	2012	121	Painset et al. 2019	False	95,97	3049500	5	3049500	3111476	SRR7440580
RL15000411	Milk and milk products	Bovine Cheese category not specified	FR	2011	121	Painset et al. 2019	False	95,82	3002795	5	3002795	3066261	SRR7440882
RL15000413	Milk and milk products	Unspecified Melted cheese	FR	2011	121	Painset et al. 2019	False	95,93	3078606	4	3078606	3140222	SRR7440884
RL15000456	Milk and milk products	Sheep Cheese category not specified	SK	2012	121	Painset et al. 2019	False	94,91	2879675	4	2879675	2882088	SRR7440736
RL15000462	Meat and meat products	Unspecified Deli product - Sausage	FR	2011	121	Painset et al. 2019	false	95,35	2937240	234	2937240	3061543	SRR7441184
RL15000466	Meat and meat products	Poultry not specified Deli product - Other product	FR	2011	121	Painset et al. 2019	False	95,69	2998049	10	2998049	3064705	SRR7441188
RL15000469	Meat and meat products	Swine Deli product - Other product	FR	2011	121	Painset et al. 2019	False	95,78	2960137	4	2960137	3023320	SRR7441168
RL15000470	Meat and meat products	Swine Deli product - Other product	FR	2011	121	Painset et al. 2019	False	95,75	2959334	6	2959334	3023084	SRR7441278
RL15000471	Meat and meat products	Swine Deli product - Other product	FR	2011	121	Painset et al. 2019	False	95,74	2960079	6	2960079	3023803	SRR7440938
RL15000472	Meat and meat products	Swine Deli product - Other product	FR	2011	121	Painset et al. 2019	False	95,63	2959841	7	2959841	3024112	SRR7441251
RL15000484	Meat and meat products	Swine Deli product - Sausage	FR	2011	121	Painset et al. 2019	False	95,92	3001820	9	3001820	3064738	SRR7440755
RL15000485	Meat and meat products	Gallus gallus (fowl) Deli products - Pate	FR	2011	121	Painset et al. 2019	False	95,28	2995759	6	2995759	3059555	SRR7441279

RL15000488	Meat and meat products	Unspecified Deli products - Pate	FR	2011	121	Painset et al. 2019	False	95,64	2962852	6	2962852	3028424	SRR7441293
RL15000494	Meat and meat products	Unspecified Deli product - Sausage	FR	2012	121	Painset et al. 2019	False	95,07	2960858	6	2960858	3022782	SRR7441048
RL15000495	Meat and meat products	Swine Deli product - Sausage	FR	2012	121	Painset et al. 2019	False	95,87	3032968	13	3032968	3100060	SRR7441179
RL15000496	Meat and meat products	Unspecified Deli products - Pate	FR	2012	121	Painset et al. 2019	False	95,81	2959345	6	2959345	3023224	SRR7441157
RL15000498	Meat and meat products	Unspecified Deli products - Pate	FR	2012	121	Painset et al. 2019	False	95,69	3026911	4	3026911	3088527	SRR7441110
RL15000499	Meat and meat products	Unspecified Deli product - Sausage	FR	2012	176	Painset et al. 2019	False	95,27	2963718	5	2963718	3027073	SRR7440553
RL15000501	Meat and meat products	Unspecified Deli product - Sausage	FR	2012	121	Painset et al. 2019	False	95,83	2997932	8	2997932	3063996	SRR7440799
RL15000516	Meat and meat products	Unspecified Meat - Cut Cooked	DN	2011	121	Painset et al. 2019	False	95,02	3021076	6	3021076	3084908	SRR7440870
RL15000517	Meat and meat products	Unspecified Meat - Cut Cooked	DN	2011	121	Painset et al. 2019	false	95,03	3018917	21	3018917	3088480	SRR7440873
RL15000526	Meat and meat products	Unspecified Meat - Cut Sliced	DN	2012	121	Painset et al. 2019	False	95,89	3033380	5	3033380	3096717	SRR7440672
RL15000532	Meat and meat products	Unspecified Meat - Cut Sliced	DN	2011	121	Painset et al. 2019	False	95,01	2933685	4	2933685	2995301	SRR7441231
RL15000660	Meat and meat products	unspecifed (spain)	ES	2010	121	Painset et al. 2019	False	95,99	3032479	4	3032479	3094095	SRR7440717
RL15000661	Meat and meat products	Swine Deli product - Ham Smoked	ES	2010	121	Painset et al. 2019	False	95,98	3032701	5	3032701	3094681	SRR7440639
RL15000664	Meat and meat products	Gallus gallus (fowl) Deli product - Other product	ES	2011	121	Painset et al. 2019	False	95,98	3034902	5	3034902	3098339	SRR7440852
RL15000667	Meat and meat products	Swine Deli product - Sliced Cooked	ES	2011	121	Painset et al. 2019	False	95,91	3034176	5	3034176	3095960	SRR7440653
RL15000668	Fish and fishery products	Fish Smoked (Salmon)	ES	2011	121	Painset et al. 2019	False	96,04	3032923	10	3032923	3099223	SRR7440658
RL15000671	Fish and fishery products	Fish Smoked (Salmon)	ES	2011	121	Painset et al. 2019	False	95,99	3067918	14	3067918	3142084	SRR7440555
RL15000674	Fish and fishery products	Fish Smoked (Salmon)	ES	2011	121	Painset et al. 2019	False	95,98	3076276	6	3076276	3139994	SRR7440566

RL15000675	Fish and fishery products	Fish Smoked (Salmon)	ES	2011	121	Painset et al. 2019	False	95,85	3040488	9	3040488	3106467	SRR7441029
RL15000682	Meat and meat products	Geese Deli product - Sliced Cooked	ES	2011	121	Painset et al. 2019	False	95,93	3031198	6	3031198	3094988	SRR7441178
RL15000685	Fish and fishery products	Fish Smoked (Salmon)	ES	2011	121	Painset et al. 2019	False	96	3036488	3	3036488	3097774	SRR7441310
RL15000687	Meat and meat products	Swine Deli product - Sliced Cooked	ES	2011	121	Painset et al. 2019	False	95,97	2987536	27	2987536	3065948	SRR7441109
RL15000689	Fish and fishery products	Fish Smoked (Salmon)	ES	2011	121	Painset et al. 2019	False	95,05	2933107	6	2933107	2995229	SRR7441045
RL15000692	Fish and fishery products	Fish Smoked	ES	2011	121	Painset et al. 2019	False	95,83	3039863	10	3039863	3114518	SRR7441111
RL15000693	Fish and fishery products	Fish Gravad/slightly salted (Salmon)	ES	2011	121	Painset et al. 2019	False	95,88	3042661	9	3042661	3115994	SRR7441307
RL15000705	Meat and meat products	Gallus gallus (fowl) Meat - Cut Cooked	ES	2010	121	Painset et al. 2019	False	95,67	2987373	7	2987373	3052632	SRR7441194
RL15000707	Composite dishes	Mixed sources Sandwich	ES	2010	121	Painset et al. 2019	False	95,96	3032981	3	3032981	3094267	SRR7440731
RL15000709	Composite dishes	Mixed sources Ready made meal	ES	2010	121	Painset et al. 2019	False	94,98	2933591	61	2933591	3016426	SRR7440763
RL15000712	Meat and meat products	Swine Deli product - Sausage	ES	2010	121	Painset et al. 2019	False	95,94	3015712	12	3015712	3085411	SRR7440620
RL15000713	Meat and meat products	Swine Deli product - Sausage	ES	2010	121	Painset et al. 2019	False	95,92	3018512	8	3018512	3084638	SRR7440729
RL15000718	Composite dishes	Mixed sources Ready made meal	ES	2010	121	Painset et al. 2019	False	95,35	2959139	3	2959139	3020410	SRR7441334
RL15000720	Meat and meat products	Unspecified Meat - Minced	ES	2011	121	Painset et al. 2019	False	95,95	3033583	6	3033583	3097412	SRR7440866
RL15000724	Composite dishes	Mixed sources Ready to eat salad	ES	2011	121	Painset et al. 2019	False	96,11	2985295	15	2985295	3055080	SRR7440868
RL15000731	Fish and fishery products	Fish Smoked (Salmon)	CZ	2010	121	Painset et al. 2019	False	95,91	3093884	7	3093884	3095946	SRR7441075

RL15001299	Meat and meat products	Bovine Meat - Cut	UK	2010	121	Painset et al. 2019	False	96,04	3083146	4	3083146	3144675	SRR7440549
RL15001300	Meat and meat products	Poultry not specified Deli product - Other product	UK	2010	121	Painset et al. 2019	False	95,89	3049492	16	3049492	3115903	SRR7440539
RL15001301	Meat and meat products	Swine Deli product - Ham	UK	2011	121	Painset et al. 2019	False	95,36	3124574	7	3124574	3194162	SRR7440540
RL15001981	Fruit, vegetables, cereals and herbs	Vegetal Vegetable	UK	2010	121	Painset et al. 2019	False	95,7	2964659	5	2964659	3026278	SRR7441191
SE-FI-F-FF-115	Fish and fishery products	Gravad Salmon - - Distributed by Denmark - fish	SE	2010	121	Felix et al. 2020	False	95,68	2994288	2	2994288	3055294	ERS4774958 (SAMEA7007130)
SE-FI-F-FF-24	Fish and fishery products	Gravad Salmon - - Sweden - fish	SE	2010	121	Felix et al. 2020	False	94,88	2912456	2	2912456	2973523	ERS4774962 (SAMEA7007134)
SE-FI-F-FF-29	Fish and fishery products	Gravad Salmon - - Sweden - fish	SE	2010	121	Felix et al. 2020	False	95,5	3094363	4	3094363	3095712	ERS4774963 (SAMEA7007135)
SE-FI-F-FF-30	Fish and fishery products	Gravad Salmon - - Sweden - fish	SE	2010	121	Felix et al. 2020	False	95,01	3011698	1	3011698	3011698	ERS4774964 (SAMEA7007136)
SE-FI-F-SM-18	Fish and fishery products	Cold smoked trout - - Sweden - fish	SE	2010	121	Felix et al. 2020	False	95,57	3066231	1	3066231	3066231	ERS4774971 (SAMEA7007143)
SE-FI-F-SM-22	Fish and fishery products	Cold smoked salmon - - Sweden - fish	SE	2010	121	Felix et al. 2020	False	94,67	2914096	111	2914096	3004762	ERS4774972 (SAMEA7007144)
SE-FI-F-SM-26	Fish and fishery products	Cold smoked salmon - - Sweden - fish	SE	2010	121	Felix et al. 2020	False	95,66	3004201	3	3004201	3065789	ERS4774975 (SAMEA7007147)
SE-FI-F-SM-28	Fish and fishery products	Cold smoked salmon - - Sweden - fish	SE	2010	121	Felix et al. 2020	False	95,49	3064940	29	3064940	3072350	ERS4774977 (SAMEA7007149)
SE-FI-F-SM-33	Fish and fishery products	Cold smoked salmon - - Sweden - fish	SE	2010	121	Felix et al. 2020	False	95,02	3039919	5	3039919	3041647	ERS4774979 (SAMEA7007151)

SE-FI-F-SM-35	Fish and fishery products	Cold smoked salmon - - Sweden - fish	SE	2010	121	Felix et al. 2020	False	94,91	2974219	1	2974219	2974219	ERS4774980 (SAMEA7007152)
SE-FI-F-SM-36	Fish and fishery products	Cold smoked salmon - - Sweden - fish	SE	2010	121	Felix et al. 2020	false	96,53	3071544	120	3071544	3226349	#N/A
SE-FI-F-SM-39	Fish and fishery products	Gravad Salmon - - Sweden - fish	SE	2010	121	Felix et al. 2020	False	94,94	3016306	1	3016306	3016306	ERS4774983 (SAMEA7007155)
SE-FI-F-SM-41	Fish and fishery products	Cold smoked salmon - - Sweden - fish	SE	2010	121	Felix et al. 2020	False	95,49	3062956	2	3062956	3063568	ERS4774985 (SAMEA7007157)
SE-FI-F-SM-43	Fish and fishery products	Cold smoked salmon - - Sweden - fish	SE	2010	121	Felix et al. 2020	False	94,91	2912950	4	2912950	2974755	ERS4774987 (SAMEA7007159)
SE-PE-FI-M-52	Processing environment	- Slicer, transportingband - Sweden - fish	SE	2010	121	Felix et al. 2020	False	95,49	3004526	2	3004526	3065623	ERS4774996 (SAMEA7007168)
SE-PE-FI-M-80	Processing environment	- Trolley nr 50 (cold smoked) - Sweden - fish	SE	2014	121	Felix et al. 2020	False	95,62	3056157	2	3056157	3056438	ERS4774997 (SAMEA7007169)
SE-PE-FI-M-90	Processing environment	- Transport band - Sweden - fish	SE	2014	121	Felix et al. 2020	False	95,62	3003100	22	3003100	3069192	ERS4774998 (SAMEA7007170)
SE-PE-ME-D-84	Processing environment	- Drain price room - Sweden - meat	SE	2014	121	Felix et al. 2020	False	95,33	3062460	24	3062460	3068897	ERS4775004 (SAMEA7007176)
SE-PE-ME-M-66	Processing environment	- chopping room/chopping machine - Sweden - meat	SE	2010	121	Felix et al. 2020	False	95,55	3061196	7	3061196	3063486	ERS4775005 (SAMEA7007177)
SE-PE-ME-M-69	Processing environment	- Transport band to slicing machine - Sweden - meat	SE	2010	121	Felix et al. 2020	False	95	2950730	17	2950730	3017608	ERS4775007 (SAMEA7007179)
SE-PE-ME-M-73	Processing environment	- Sausagefiller - Sweden - meat	SE	2014	121	Felix et al. 2020	False	95,52	3061321	5	3061321	3062865	ERS4775009 (SAMEA7007181)
SE-PE-ME-M-74	Processing environment	- transport band going in - Sweden - meat	SE	2014	121	Felix et al. 2020	False	95,46	3036400	1	3036400	3036400	ERS4775010 (SAMEA7007182)
SE-PE-ME-M-81	Processing environment	- Paté chopping machine - Sweden - meat	SE	2014	121	Felix et al. 2020	False	95,24	2970063	19	2970063	3035515	ERS4775015 (SAMEA7007187)
SE-PE-ME-M-89	Processing environment	- Blade - Sweden - meat	SE	2014	121	Felix et al. 2020	False	95,58	3095587	29	3095587	3105868	ERS4775021 (SAMEA7007193)
SE-PE-ME-S-58	Processing environment	- Floor drain - ham polishing - Sweden - meat	SE	2010	236	Felix et al. 2020	False	95,29	3025634	10	3025634	3028631	ERS4775028 (SAMEA7007200)
SI-FAR-WT-51	Soil & farm environment	Environnement agricole eau	SI	2008	121	Typelipo	False	95,47	3083305	3	3083305	3084036	ERS4775061 (SAMEA7007233)

SLCC2216	animal	unknow	FR	1964	121	seelinger collection	False	94,59	2905441	10	2905441	2971357	#N/A
SLCC3296	animal	unknow	DE	1970	121	seelinger collection	False	95,47	2953689	8	2953689	3019949	#N/A
SLCC3306	animal	unknow	DE	1970	121	seelinger collection	False	94,1	2871603	14	2871603	2939436	#N/A
SRR6116310	Farm animals	bovine brain	US	2017	121	Pirone-Davies et al. 2018	False	95,32	2964303	1	2964303	2964303	SRR6116310
SRR6116326	Farm animals	bovine abortion	US	2017	121	Pirone-Davies et al. 2019	False	95,45	2923380	1	2923380	2923380	SRR6116326
UK-VE-U-UN-93	vegetable	#N/A	UK	2018	121	Felix et al. 2020	False	95,63	3028446	7	3028446	3030249	ERS4775197 (SAMEA7007369)
UK-OTH-FE-U5	Wild animals	Hedgehog	UK	ND	121	Felix et al. 2020	False	95,78	3030228	6	3030228	3095274	#N/A
